# Genome-wide association study of REM sleep behavior disorder in Parkinson’s disease

**DOI:** 10.1038/s41531-025-01078-w

**Published:** 2025-09-25

**Authors:** Yuri L. Sosero, Karl Heilbron, Pierre Fontanillas, Lucy Norcliffe-Kaufmann, Eric Yu, Uladzislau Rudakou, Jennifer A. Ruskey, Kathryn Freeman, Farnaz Asayesh, Kajsa A. Brolin, Maria Swanberg, Huw R. Morris, Lesley Wu, Raquel Real, Lasse Pihlstrøm, Manuela Tan, Thomas Gasser, Kathrin Brockmann, Hui Liu, Michele T. M. Hu, Donald G. Grosset, Simon J. G. Lewis, John B. Kwok, Pau Pastor, Ignacio Alvarez, Matej Skorvanek, Alexandra Lackova, Miriam Ostrozovicova, Mie Rizig, 23andMe Research Team, The International Parkinson’s Disease Genomics Consortium, Lynne Krohn, Ziv Gan-Or

**Affiliations:** 1 Department of Human Genetics, McGill University, Montréal, QC Canada; 2 The Neuro (Montreal Neurological Institute-Hospital), McGill University, Montréal, QC Canada; 3 23andMe, Inc., Sunnyvale, CA USA; 4 Lund University, Translational Neurogenetics Unit, Department of Experimental Medical Science, Lund, Sweden; 5 Department of Clinical and Movement Neurosciences, UCL Queen Square Institute of Neurology, University College London, London, UK; 6 UCL Movement Disorders Centre, University College London, London, UK; 7 Department of Neurology, Oslo University Hospital, Oslo, Norway; 8 Department of Neurodegeneration at Hertie Institute for Clinical Brain Research, Tuebingen, Germany; 9 Oxford Parkinson’s Disease Centre (OPDC), University of Oxford, Oxford, UK; 10 Nuffield Department of Clinical Neurosciences, University of Oxford, Oxford, UK; 11 Institute of Neurological Sciences, Queen Elizabeth University Hospital, Glasgow, UK; 12 Parkinson’s Disease Research Clinic, Brain and Mind Centre, School of Medical Sciences, University of Sydney, Sydney, NSW Australia; 13 Unit of Neurodegenerative Diseases, Department of Neurology, University Hospital Germans Trias i Pujol and The Germans Trias i Pujol Research Institute (IGTP) Badalona, Barcelona, Spain; 14 Department of Neurology, Hospital Universitari Mutua de Terrassa, Barcelona, Spain; 15 Department of Neurology, Pavol Jozef Šafárik University in Košice, Košice, Slovakia; 16 Department of Neurology, University Hospital of L. Pasteur, Kosice, Slovakia; 17 UCL Queen Square Institute of Neurology, London, UK; 18 Department of Neurology and Neurosurgery, McGill University, Montreal, QC Canada

**Keywords:** Diseases, Genetics, Neurology, Neuroscience

## Abstract

REM sleep behavior disorder (RBD), is a prodromal synucleinopathy affecting a subset of Parkinson’s disease (PD) patients and associated with neuropsychiatric symptoms. This study compared the genetic profiles of 13,020 PD patients with probable RBD (PD + RBD) and 5403 without (PD-RBD) using genome-wide association study (GWAS). RBD was assessed by questionnaires or self-reporting. Potential genetic correlations between neuropsychiatric traits and PD + RBD were assessed using linkage disequilibrium score regression. The top variant in the *SNCA* locus was associated with PD + RBD (rs10005233-T, OR = 1.21, 95% CI = 1.16–1.27, *p* = 1.81e−15). PD risk variants in *SNCA* (rs5019538-G, OR = 0.85, 95% CI = 0.81–0.89, *p* = 2.46e−10; rs356182-G, OR = 0.89, 95% CI = 0.84–0.95, *p* = 0.0001) and *LRRK2* loci (rs34637584, OR = 0.41, 95% CI = 0.28–0.61, *p* = 1.04e−5) were associated with reduced PD + RBD risk. A suggestive genetic correlation between attention deficit hyperactivity disorder and PD + RBD was observed but was not statistically significant after correction. These findings highlight genetic distinctions between PD + RBD and PD-RBD, offering insights into PD stratification and potential subtype-specific treatments.

## Introduction

Rapid-eye-movement (REM) sleep behavior disorder (RBD) is a parasomnia characterized by the absence of muscle atonia during REM sleep and dreams enactment^1^. When no neurological conditions or other concomitant factors are identified, it is referred to as isolated/idiopathic RBD (iRBD)^2^. iRBD is typically considered a prodromal stage of synucleinopathies, as about 80%–90% of the cases convert to either Parkinson’s disease (PD), dementia with Lewy bodies (DLB) or, more rarely, multiple system atrophy (MSA)^3,4^. These disorders are all characterized by the accumulation of alpha-synuclein, encoded by the *SNCA* gene^5^. RBD is therefore a key prodromal clinical marker of synucleinopathies, and its presence is also associated with a distinctive, more severe clinical presentation. In PD patients with RBD (approximately 25–58% of cases^6^), RBD is associated with a more malignant phenotype, characterized by faster progression^7^ and greater frequency and/or severity of neuropsychiatric manifestations, including cognitive decline, hallucinations, depression, anxiety and apathy^8–11^. RBD can occur before (then it will be referred to as iRBD), or after the onset of PD and typically PD + RBD cohorts include similar frequencies of individuals who had RBD before and after the onset of RBD^6^.

In recent years, it was shown that the genetic background of iRBD only partially overlaps with that of PD or DLB. Genes such as *GBA1*^12^, *TMEM175*^13^ and *SNCA*^14^ are important across all conditions^15,16^, whereas other genes including *LRRK2*^17^, *APOE*^18^ and familial PD genes^19^, seem to not have a major role in iRBD. A recent RBD genome-wide association study (GWAS) identified 5 risk loci associated with RBD^20^, namely *GBA1, TMEM175, INPPSF, SNCA* and *SCARB2*. Notably, the variants associated with RBD in the *SNCA* and *SCARB2* regions were different and independent to those associated with PD^15,20^, supporting RBD as a distinctive subtype, with specific genetic and clinical correlates.

In the current study, we aimed to examine whether there are genetic differences between these two sub-groups of patients: PD patients with probable RBD (PD + RBD) and PD patients without RBD (PD-RBD). We used a case-only design, wherein 15 PD cohorts with available data on probable RBD (Table 1) were divided into these two sub-groups of patients. In total, the study included 18,423 patients, composed of 5403 PD + RBD patients and 13,020 PD-RBD patients. To further explore the relationships between RBD and neuropsychiatric manifestations in PD, we performed genetic correlation and Mendelian randomization (MR) analyses using the GWAS summary statistics of the current study and multiple neuropsychiatric conditions.

**Table 1:** Demographic characteristics of PD patients in the individual cohorts

Center	PD + RBD, *n*	Age PD + RBD (SD)	%Fem PD + RBD	PD-RBD, n	Age PD-RBD (SD)	%Fem PD-RBD	Total
Oslo	130	65.6 (8.8)	25%	180	66.1 (10)	44%	310
Lund	365	71.4 (7)	39%	555	70.8 (9.2)	33%	920
McGill	285	67.4 (9.2)	31%	217	67 (8.8)	49%	502
AMP-PD	111	66.4 (9.4)	69%	269	65.5 (10.3)	62%	380
Sydney	105	59 (10.8)	32%	125	60 (10.7)	38%	230
Tuebingen	453	68.8 (9.1)	33%	659	67.7 (10.2)	38%	1112
Barcelona	133	69.5 (9.8)	44%	71	74.3 (10)	66%	204
PRoBaND	585	67.3 (8.9)	30%	1134	67.7 (9.3)	38%	1719
PFP	257	62.4 (12.5)	37%	339	61.4 (13)	47%	596
OPDC	274	67.3 (9.3)	27%	539	67.4 (9.6)	38%	813
Kosice	102	71.4 (8.3)	33%	225	69.2 (10.7)	42%	327
23andMe	2603	NA^a^	38%	8707	NA^a^	45%	11,310
Total	5403	/	/	13,020	/	/	18,423

*PD* *+* *RBD, n* number of Parkinson’s disease patients with REM sleep behavior disorder, *PD-RBD, n* number of Parkinson’s disease patients without REM sleep behavior disorder, *Age* mean age in the group, *SD* standard deviation, *%Fem* percentage of females, *Tot* total number of PD patients in the cohort, *Oslo* Oslo University Hospital, *Lund* Lund University, *McGill* McGill University, *AMP-PD* Accelerating Medicines Partnership Parkinson’s disease, including the New Discovery of Biomarkers (BioFIND), the Harvard Biomarker Study (HBS) and the Parkinson’s Disease Biomarkers Program (PDBP) cohorts, *Sydney* University of Sydney, *Tuebingen* University of Tuebingen, *Barcelona* Hospital Universitari Mutua de Terrassa, *PRoBaND* Parkinson’s repository of biosamples and networked datasets, *PFP* Parkinson’s Families Project, *OPDC* Oxford Parkinson’s Disease Centre, *Kosice* Pavol Jozef Šafárik University in Kosice.

^a^23andMe only provides age ranges (i.e., in cases: 5 individuals <30 years of age, 49 individuals 30–45, 346 individuals 45–60, 2203 individuals >60; in controls: 11 individuals <30, 146 individuals 30–45, 1302 individuals 45–60, 7248 individuals >60).

## Results

### Genome-wide association study identifies the *SNCA* and *LRRK2* loci as modifiers of risk for RBD in PD

To assess whether genetics can affect the risk of RBD in PD we performed GWAS between PD + RBD (*N* = 5403) and PD-RBD (*N* = 13,020). We evaluated the genomic inflation using quantile-quantile plots (Q-Q plots) and the lambda factor, showing no inflation (lambda = 0.994, lambda_1000_ = 0.999) (Supplementary Fig. 1).

**Fig. 1:**
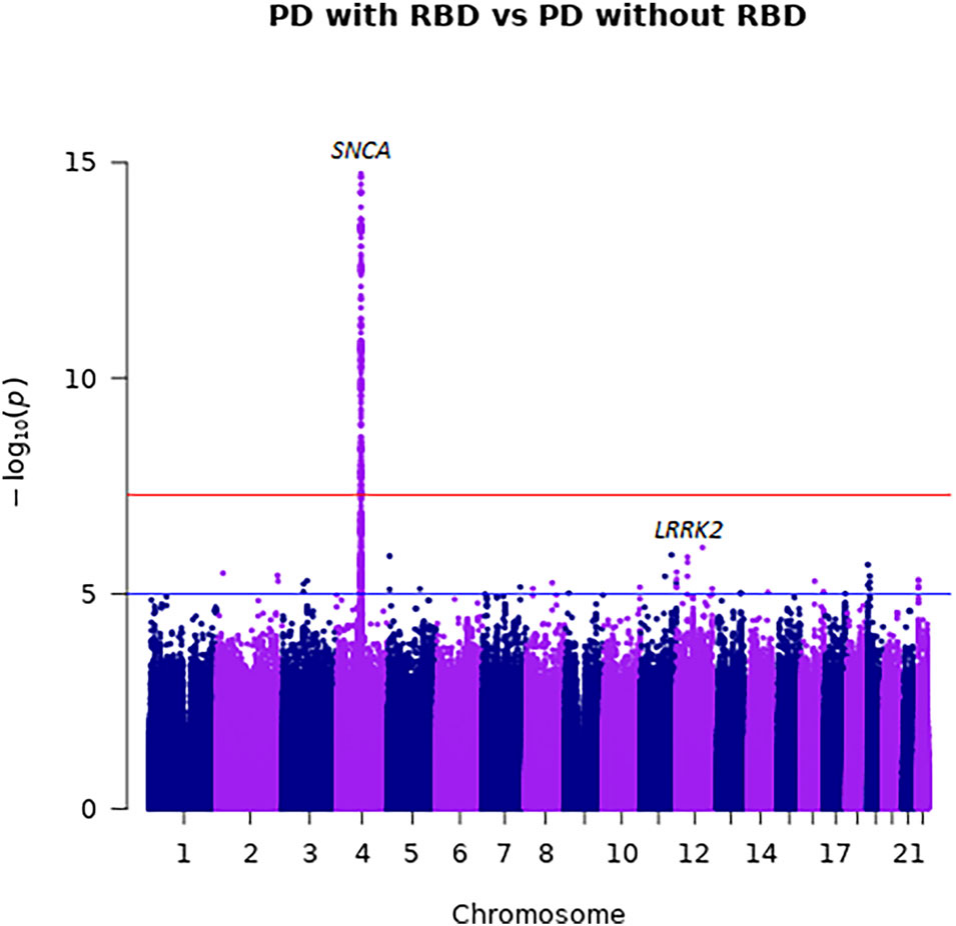
Manhattan plot of PD with RBD vs. PD without RBD. A Manhattan plot showing the results of the GWAS meta-analysis, comparing PD with RBD and PD without RBD, highlighting the *SNCA* and *LRRK2* loci. The *Y* axis represents the negative logarithm of *p* value, the *X* axis represents the chromosomal position of the variants and each dot on the figure represents a SNP. The red line represents the genome-wide Bonferroni-corrected statistical significance threshold (5 × 10^−8^), whereas the blue line is the false-discovery rate-corrected significance threshold (5 × 10^−5^). Chr chromosome, PD Parkinson’s disease, RBD REM sleep behavior disorder.

We found that rs10005233-T, in the 5’ region of the *SNCA* locus, was associated with PD + RBD (OR = 1.21, 95% CI = 1.16–1.27, *p* = 1.81E−15, Fig. 1). No secondary signal was detected in the GCTA-COJO analysis at a GWAS significance level. We also examined the 92 variants associated with PD in the most recently published GWAS in Europeans^15^ and Asians^21^ (Table 2 and Supplementary Table 1). Using Bonferroni correction based on the number of these variants (α/number of variants = 0.00054), we identified three associations. Two were variants in the *SNCA* locus, which were not on LD with the primary SNCA variant rs10005233-T variant nor with each other, whose minor alleles were associated with decreased risk for PD + RBD (rs5019538-G, OR = 0.85, 95% CI = 0.81–0.89, *p* = 2.46E−10 and rs356182-G, OR = 0.89, 95% CI = 0.84–0.95, *p* = 0.0001), and one was the LRRK2 p.G2019S variant, also associated with a reduced risk for PD + RBD (rs34637584, OR = 0.41, 95% CI = 0.28–0.61, *p* = 1.04E−5, the carrier frequency for this variant across the different cohorts is detailed in Table 3). These three variants were associated with increased risk for PD in the most recent GWAS^15,21^. *GBA1* variants did not show significant associations with increased risk of RBD in PD (Supplementary Table 2). Additional potential associations in the *SETD1A*, *SPPL2B*, *CRHR1* and *LINC00693* loci should be further studied (Table 2).

**Table 2:** Among PD-variants, associations with RBD in PD compared to prior PD-GWAS associations

		PD with and without RBD		PD^15,37^	
Variant	Nearest gene	OR (95% CI)	*p* value	OR (95% CI)	*p* value
4:90636630	*SNCA*	0.85 (0.81–0.89)	2.46E−10^a^	1.17 (1.14–1.2)	1.13E−36
12:40734202	*LRRK2*	0.41 (0.28–0.61)	1.04E−05^a^	11.35 (9.44–13.63)	3.61E−148
4:90626111	*SNCA*	0.89 (0.84–0.95)	0.0001365^a^	1.32 (1.29–1.35)	3.89E−154
16:30977799	*SETD1A*	0.92 (0.88–0.97)	0.001952	1.09 (1.07–1.12)	5.12E−20
19:2341047	*SPPL2B*	1.09 (1.03–1.15)	0.002506	0.93 (0.91–0.95)	4.18E−10
17:43798308	*CRHR1*	1.19 (1.05–1.36)	0.008267	0.79 (0.75–0.84)	6.71E−16
3:28705690	*LINC00693*	0.95 (0.9–0.99)	0.02552	1.07 (1.05–1.09)	8.09E−12

*PD* Parkinson’s disease, *RBD* REM sleep behavior disorder, *OR (95% CI)* odds ratio with relative 95% confidence interval.

^a^Variant statistically significant after Bonferroni correction (*α*/number of variants = 0.00054).

**Table 3:** Carriers of the *LRRK2* p.G2019S variant across different cohorts

Cohort	Non-carriers PD-RBD, *N*	Carriers PD-RBD, *N* (%)	Non-carriers PD + RBD, *N*	Carriers PD + RBD, *N* (%)
Oslo	156	1 (0.64%)	122	1 (0.81%)
Lund	552	3 (0.54%)	364	1 (0.27%)
McGill	212	5 (2.30%)	282	3 (1.05%)
AMP-PD	258	9 (3.37%)	110	1 (0.90%)
Sydney	115	1 (0.86%)	98	1 (1.01%)
Tuebingen	365	0 (0.00%)	641	0 (0.00%)
Barcelona	45	0 (0.00%)	100	0 (0.00%)
PRoBaND	1132	2 (0.18%)	582	3 (0.51%)
PFP	328	9 (2.67%)	246	6 (2.38%)
OPDC	537	2 (0.37%)	274	0 (0.00%)
Kosice	216	1 (0.46%)	101	0 (0.00%)
23andMe	8459	248 (2.85%)	2584	19 (0.73%)
Total^a^	12,010	281 (2.29%)	4863	35 (0.71%)

*PD-RBD* participants without REM sleep behavior disorder (RBD), *PD* *+* *RBD* participants with RBD, *N* number of participants, *%* percentage of participants, *Oslo* Oslo University Hospital, *Lund* Lund University, *McGill* McGill University, *AMP-PD* Accelerating Medicines Partnership Parkinson’s disease, including the New Discovery of Biomarkers (BioFIND), the Harvard Biomarker Study (HBS) and the Parkinson’s Disease Biomarkers Program (PDBP) cohorts, *Sydney* University of Sydney, *Tuebingen* University of Tuebingen, *Barcelona* Hospital Universitari Mutua de Terrassa, *PRoBaND* Parkinson’s repository of biosamples and networked datasets, *PFP* Parkinson’s Families Project, *OPDC* Oxford Parkinson’s Disease Centre, *Kosice* Pavol Jozef Šafárik University in Kosice.

^a^The total excludes individuals with unknown carrier status for p.G2019S.

### Genetic correlation and causative associations between PD with RBD and neuropsychiatric disorders

**Fig. 2:**
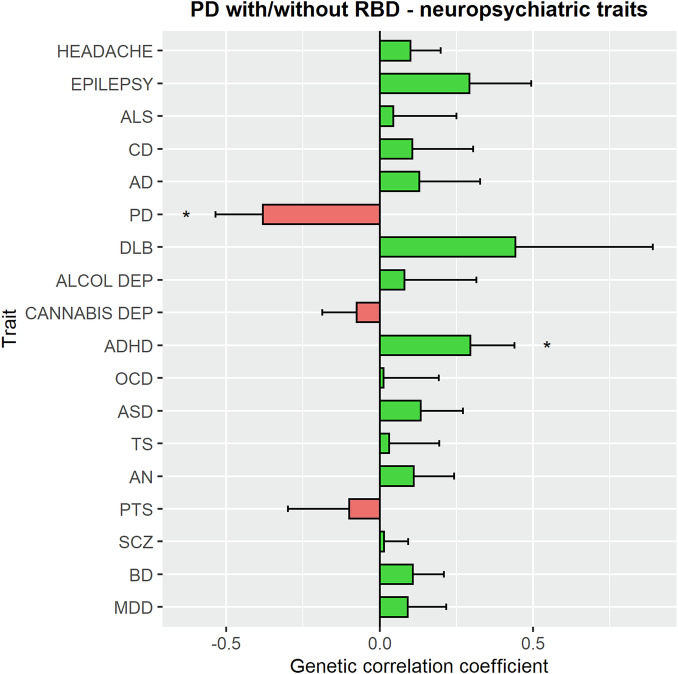
Genetic correlation between PD with RBD and neuropsychiatric traits. The bar plot shows the genetic correlations between PD with RBD and neuropsychiatric traits. The correlation coefficient is illustrated on the *X* axis. Green bars represent positive correlations whereas red bars negative ones (i.e., a positive correlation of the neuropsychiatric trait with PD without RBD). The asterisks highlight the nominally significant correlations. ALS amyotrophic lateral sclerosis, CD cognitive decline, AD Alzheimer’s disease, PD Parkinson’s disease, DLB dementia with Lewy bodies, Alcohol dep alcohol dependence, cannabis dep cannabis dependence, ADHD attention deficit hyperactivity disorder, OCD obsessive-compulsive disorder, ASD autism spectrum disorder, TS Tourette syndrome, AN anorexia nervosa, PTS post-traumatic syndrome, SCZ schizophrenia, BD bipolar disorder, MDD major depressive disorder.

To examine potential genetic correlations between the risk of RBD in PD and multiple neuropsychiatric conditions, we performed LDSC (Fig. 2 and Supplementary Table 3). We found that genetic factors associated with the presence of RBD in PD are mildly genetically correlated with attention deficit hyperactivity disorder (ADHD, rg = 0.30, SE = 0.14, *p* = 0.04). The most recently published European PD GWAS was negatively genetically correlated with PD + RBD (rg = −0.38, SE = 0.15, *p* = 0.01). However, these correlations were not statistically significant after Bonferroni correction (*α* = 0.0025).

To assess possible causative associations between neuropsychiatric conditions and PD + RBD we performed MR using neuropsychiatric disorders as exposures and PD + RBD as the outcome (Supplementary Figs. 2 and 3 and Supplementary Tables 4–7). No test showed a statistically significant causative association between neuropsychiatric traits and PD + RBD. However, our power for this analysis was suboptimal (35.7%), therefore there could be associations that we could not detect. We were not able to conduct reverse MR using PD + RBD as the exposure since only one locus passed GWAS significance, preventing us from performing appropriate sensitivity analyses.

## Discussion

In the current GWAS, we found that variants in the *SNCA* and *LRRK2* loci may be associated with the risk of RBD in PD. Additional loci (*SETD1A*, *SPPL2B*, *CRHR1* and *LINC00693*) require further studies to examine whether they have a role in PD + RBD. The top variant in the *SNCA* locus, rs10005233-T, was previously reported to be associated with iRBD in a candidate gene study (OR = 1.43, 95% CI = 1.27–1.62, *p* = 1.1e−08)^14^. Another study, using the Oslo and Parkinson’s Progression Marker Initiative cohorts, found another variant in the *SNCA* locus associated with PD + RBD (rs3756063), which is in strong linkage disequilibrium (LD) with rs10005233-T (*D*’ = 0.97, *r*^2^ = 0.91)^22^. Furthermore, rs10005233-T is in LD with other 5’ region *SNCA* variants associated with synucleinopathies, including rs7681440 (*D*’ = 0.99, *r*^2^ = 0.94), associated with DLB^23^, rs763443 (*D*’ = 0.89, *r*^2^ = 0.78), a secondary PD GWAS signal^14,15,24^, as well as rs2583988 (*D*’ = 0.99, *r*^2^ = 0.40), a variant located in the *SNCA-AS1* region (discussed below) and associated with Lewy body variant of Alzheimer’s disease (ADLBV)^25^. It is still unclear whether it is a specific variant in the *SNCA* locus or the presence of a specific *SNCA* haplotype that drives these associations with cognitive phenotypes across synucleinopathies^14^. The rs10005233 variant is also in LD (*D*’ = 0.97, *r*^2^ = 0.91) with the top signal of a recently published RBD GWAS, rs3756059^20^, which was associated with reduced expression of *SNCA-AS1*, an antisense RNA molecule that could potentially reduce the translation of alpha-synuclein when it is overexpressed or increase the translation of alpha-synuclein when it is down-regulated. Notably, this reduced expression of *SNCA-AS1* is mainly in cortical areas^20^, thus potentially increasing alpha-synuclein levels and exposing the cerebral cortex to a greater risk of neurodegeneration in carriers of this RBD-associated variant. The latter hypothesis should be tested in relevant animal models. Altogether, these data suggest that, depending on possible region-specific effects, different *SNCA* variants might play different roles in synucleinopathies.

We found that three of the 92 PD GWAS signals associated with increased PD risk in Europeans and Asians^15,21,26^, the LRRK2 variant p.G2019S and the SNCA variants rs5019538 and rs356182, were less frequent in PD + RBD compared to PD-RBD. The association between p.G2019S and PD-RBD is in line with a previously reported reduced frequency of RBD in PD patients carrying this variant (OR = 0.49, 95% CI 0.39–0.61, *p* < 0.001)^27,28^, and with lack of p.G2019S carriers in about 1000 iRBD patients in another study^19^. In addition to a reduced occurrence of RBD, carriers of the p.G2019S LRRK2 variant also present an overall more benign phenotype, including less frequent and milder cognitive decline^29,30^. These findings, together with the negative correlation between the presence of RBD in PD and the most recent PD GWAS in Europeans^15^ may suggest that overall, the PD-RBD subgroup is more genetically similar to PD than the subgroup of PD + RBD. However, the latter group may still also be genetically similar to PD in general, and in order to perform proper genetic correlation between PD + RBD, PD-RBD and PD, each of these groups should be compared to separate control groups, followed by re-analyses of genetic correlations.

These findings further support a pathophysiological relationship between the manifestation of RBD in PD and cognitive decline, which is in line with the comorbidity of these two clinical entities. The rs10005233-T *SNCA* variant is associated with both RBD and increased cognitive decline, while the LRRK2 p.G2019S variant is associated with reduced risk of RBD and reduced cognitive decline. These findings may suggest a potential pathophysiological relationship between the manifestation of RBD in PD and cognitive decline, which at least in part is affected by genetics. It was hypothesized that this clinical and pathophysiological correlation could reflect the two alternative directions of alpha-synuclein spreading, body-first or brain-first^31^. In body-first PD, alpha-synuclein pathology may start in the enteric nervous system, whereas in brain-first PD it may arise in the amygdala, entorhinal cortex and substantia nigra. These different neuropathological patterns correspond to two different subgroups of clinical progression. In body-first PD, RBD may manifest before the motor PD symptoms, and cognitive decline occurs faster, whereas in brain-first PD, RBD may occur after the onset of motor PD symptoms, if at all, and cognitive impairment develops more slowly^31–34^. We can therefore speculate that the *SNCA* rs10005233 variant associated with PD + RBD might also be associated with the body-first subtype of PD, whereas the LRRK2 p.G2019S variant might be associated with the brain-first PD subtype, with less frequent RBD and milder cognitive decline. Since we cannot determine in our data which patients had RBD prior to PD diagnosis and which had it after PD diagnosis, this hypothesis should be studied in future genetic analyses of brain-first vs. body-first PD.

Similar to previous reports in iRBD and PD + RBD^14,15,22^, in this study we did not observe any involvement of *APOE* variants in PD + RBD, suggesting that this gene does not affect RBD risk in PD patients. The rs117615688 variant (chromosomal position 17:43798308) in the *CRHR1* gene, located in the *MAPT* locus, was nominally associated with RBD (OR = 1.19, 95% CI = 1.05–1.36, *p* = 0.008) with an opposite direction of effect to that seen in PD (OR = 0.79, 95% CI = 0.75–0.84, *p* = 6.71E−16) (Table 2).

There are several limitations in this study. All participants were Europeans, therefore our results might not fully apply to other ancestries. We cannot completely rule out survival bias as a confounder of our results, although PD patients with and without RBD had similar age. In addition, although we included a large number of patients with PD, insufficient power in our analysis might explain the lack of causative associations between PD + RBD and neuropsychiatric traits as well as of genome-wide significance of the LRRK2 p.G2019S and *GBA1* variants. It is possible that *GBA1* variants are strongly implicated also in the PD subtype without RBD, thus counterbalancing their previously reported contribution to RBD risk^12,20^. Another limitation is represented by the inclusion of patients who developed RBD both before and after PD, as they may represent body-first vs. brain-first subtypes of PD as discussed above. Future research with larger sample sizes could investigate possible genetic and biological differences between them and specifically differentiate brain-first and body-first PD in that sense. The aim of the current study was limited to the genetic differences between PD + RBD and PD-RBD. Future studies may compare each of these sub-groups to healthy controls, or to other subgroups, and other synucleinopathies such as DLB and MSA. Such analyses may also shed more light on the genetic basis of the different synucleinopathies. Additional studies that examine gene-gene and gene-environment interactions will also provide crucial information, but such studies will require much larger sample sizes than currently available.

Since the sex distribution between PD + RBD and PD-RBD is different, sex-stratified analysis is warranted in larger, better-powered studies. Some individuals with PD-RBD could still develop RBD, which is why adjustment for age has been performed. Yet this fact could still dilute the model and the effects of specific variants. There is more than one way to interpret the findings of our study. The most straightforward interpretation is that PD + RBD and PD-RBD largely share the same genetic background, which is common to PD in general, and individual factors such as SNCA and LRRK2 variants can tip the balance to one or the other subtype. However, other scenarios are also possible, in which the different phenotypes have more distinct genetic architecture, which in the present study design we could not yet identify. This potential interpretation may be consistent with the genetic correlation analyses, but much larger studies with additional complementary genetic analyses are needed to examine this possibility. It is also possible that while some PD-associated variants are less common in PD + RBD than in PD-RBD, they are still more common in PD + RBD than in the healthy population, and this should be explored in a separate study.

In conclusion, in this study we demonstrated that the risk of PD + RBD may be modified by variants in the *SNCA* and *LRRK2* loci, and potentially other loci. These genetic associations may explain why cognitive decline is more frequently observed in PD + RBD compared to PD-RBD, with possible implications for therapeutic management of PD patients. Future research will need to further explore the relationship between genetics, biology and clinical comorbidities to define PD subtypes and implement a precision medicine guided by early markers.

## Methods

### Study design

The aim of this study is to examine whether there are differences in frequencies of genetic variants when comparing PD + RBD to PD-RBD. Such differences might highlight specific genes, variants and pathways that are more involved in one sub-type compared to the other. For this purpose, we performed a case-only GWAS in 15 cohorts with available data on Probable RBD (Table 1). We performed a GWAS in each cohort separately, comparing PD + RBD and PD-RBD, followed by a meta-analysis. Specific details on the cohort, quality control and analysis are detailed below.

### Population

The study population included 18,423 PD patients (detailed in Table 1), of whom 5403 had probable RBD (PD + RBD) and were treated as cases, whereas 13,020 did not (PD-RBD) and were treated as controls. Probable RBD was defined using either the RBD single-question screen (RBD1Q)^35^ or the RBD screening questionnaire (RBDSQ)^36^, both of which show high sensitivity and specificity in PD patients^37^. We refer to iRBD when RBD occurs prior to the neurodegeneration and to RBD for subjects with RBD regardless of the time of onset of neurodegeneration. PD was diagnosed by movement disorder specialists according to the UK Brain Bank^38^ or International Parkinson Disease and Movement Disorders Society criteria^39^. The 23andMe cohort had self-reported a diagnosis of PD as well as RBD and/or dream enactment behaviors.

The participants were of European ancestry and their clinical and genetic data were collected from 15 different cohorts (Table 1), 11 of which are from the International Parkinson’s Disease Genomics Consortium (IPDGC), three cohorts are from the Accelerating Medicines Partnership Parkinson’s disease (AMP-PD, https://amp-pd.org/) and one cohort was collected and analyzed by 23andMe Inc. (https://www.23andme.com/research/). The Central European Group on Genetics of Movement Disorders (CEGEMOD) contributed to the Kosice cohort.

### Ethical compliance statement

IRB Study Number A11-M60-21A (21-11-023) was reviewed and approved by the Research Ethics Offices (REOs). Informed written patient consent was provided in each center before the inclusion of each in the study.

### Genetic analysis

In the 23andMe cohort, participants provided informed consent and volunteered to participate in the research online, under a protocol approved by the external AAHRPP-accredited IRB, Ethical & Independent (E&I) Review Services. As of 2022, E&I Review Services is part of Salus IRB (https://www.versiticlinicaltrials.org/salusirb). DNA extraction and genotyping were performed on saliva samples by National Genetics Institute (NGI), a CLIA-licensed clinical laboratory and a subsidiary of Laboratory Corporation of America. Samples were genotyped on one of five genotyping platforms. The v1 and v2 platforms were variants of the Illumina HumanHap550 + BeadChip, including about 25,000 custom single nucleotide polymorphisms (SNPs) selected by 23andMe, with a total of about 560,000 SNPs. The v3 platform was based on the Illumina OmniExpress+ BeadChip, with custom content to improve the overlap with the 23andMe v2 array, with a total of about 950,000 SNPs. The v4 platform was a fully customized array, including a lower redundancy subset of v2 and v3 SNPs with additional coverage of lower-frequency coding variation, and about 570,000 SNPs. The v5 platform, in current use, is an Illumina Infinium Global Screening Array (~640,000 SNPs) supplemented with ~50,000 SNPs of custom content. This array was specifically designed to better capture global genetic diversity and to help standardize the platform for genetic research. Samples that failed to reach 98.5% call rate were re-analyzed. Individuals whose analyses failed repeatedly were re-contacted by 23andMe customer service to provide additional samples.

Participants were restricted to European ancestry through an analysis of local ancestry^40^. A support vector machine (SVM) to classify individual haplotypes into one of 31 reference populations was used (https://www.23andme.com/ancestry-composition-guide/). The SVM classifications are then fed into a hidden Markov model (HMM) that accounts for switch errors and incorrect assignments, and gives probabilities for each reference population in each window. Finally, we used simulated admixed individuals to recalibrate the HMM probabilities so that the reported assignments are consistent with the simulated admixture proportions. A maximal set of unrelated individuals was chosen for each analysis using a segmental identity-by-descent (IBD) estimation algorithm^41^.

We phased participant data using either an internally-developed tool, Finch (V1-V4 genotyping arrays) or Eagle2 (V5 genotyping array)^42^. Finch implements the Beagle haplotype graph-based phasing algorithm, modified to separate the haplotype graph construction and phasing steps^43^. It extends the Beagle model to accommodate genotyping error and recombination, to handle cases where there are no consistent paths through the haplotype graph for the individual being phased. We constructed haplotype graphs for European and non-European samples on each 23andMe genotyping platform from a representative sample of genotyped individuals, and then performed out-of-sample phasing of all genotyped individuals against the appropriate graph. For the X-chromosome, we built separate haplotype graphs for the non-pseudoautosomal region and each pseudoautosomal region, and these regions were phased separately.

Imputation panels created by combining multiple smaller panels have been shown to give better imputation performance than the individual constituent panels alone^44^. To that end, we combined the May 2015 release of the 1000 Genomes Phase 3 haplotypes with the UK10K imputation reference panel to create a single unified imputation reference panel^45,46^. Multiallelic sites with N alternate alleles were split into N separate biallelic sites. We then removed any site whose minor allele appeared in only one sample. For each chromosome, we used Minimac3 to impute the reference panels against each other, reporting the best-guess genotype at each site^47^. This gave us calls for all samples over a single unified set of variants. We then joined these together to get, for each chromosome, a single VCF with phased calls at every site for 6,285 samples.

In preparation for imputation, we split each chromosome of the reference panel into chunks of no more than 300,000 variants, with overlaps of 10,000 variants on each side. We used a single batch of 10,000 individuals to estimate Minimac3 imputation model parameters for each chunk^47^. We imputed phased participant data against the chunked merged reference panel using Minimac3, treating males as homozygous pseudo-diploids for the non-pseudoautosomal region. Throughout, we treated structural variants and small indels the same as SNPs.

We excluded SNPs that: (1) had a MAF < 0.01, (2) had a call rate <90%, (3) had a Hardy-Weinberg *p* < 10–20 in people with predominantly European ancestry, (4) were only genotyped on the V1 and/or V2 platforms, (5) were found on the mitochondrial chromosome or the Y-chromosome, (6) failed a test for parent-offspring transmission (specifically, we regressed the child’s allele count against the mean parental allele count and excluded SNPs with fitted <0.6 and *p* < 10–20 for a test of <1), (7) had an association with genotype date (*p* < 10–50 by ANOVA of SNP genotypes against a factor dividing genotyping date into 20 roughly equal-sized buckets), (8) had a large sex effect (ANOVA of SNP genotypes, r2 > 0.1), or (9) had probes matching multiple genomic positions in the reference genome.

We excluded SNPs with imputed r2 < 0.3, as well as SNPs that had strong evidence of a platform batch effect. For each SNP we identified the largest sub-set of the data passing other quality control criteria based on their original genotyping platform—either v2 + v3 + v4 + v5, v4 + v5, v4, or v5 only—and computed association test results for the largest passing set. The batch effect test is an *F* test from an ANOVA of the SNP dosages against a factor representing the V4 or V5 platform; we excluded results with *p* < 10–50.

Across both genotyped and imputed GWAS results, we excluded SNPs that had sample size of less than 20% of the total GWAS sample size. We also removed SNPs that did not converge during logistic regression, as identified by abs (effect) > 10 or stderr >10 on the log-odds scale. If SNPs were both genotyped and imputed, and they passed QC for both, we used results from the imputed analysis. After quality control, we had analyzed 904,040 genotyped SNPs and 25,208,208 imputed SNPs.

GWAS was performed using logistic regression adjusted for age, sex, top five principal components as well as the genotype platform to account for genotype batch effects. The significance threshold was set at *p* < 5 × 10E−8.

In the other centers, genotyping was performed using the OmniExpress, NeuroX or Global Screening (GSA) GWAS array according to the manufacturer’s instructions (Illumina Inc.). Parkinson’s Families Project (PFP) was genotyped with NeuroChip, Parkinson’s repository of biosamples and networked datasets (PRoBaND) with HumanCoreExome array, and Oxford Parkinson’s Disease Centre (OPDC) with either HumanCoreExome-12 v.1.1 or Infinium HumanCoreExome-24v.1.1 arrays. Quality control was performed following standard pipelines (detailed in https://github.com/neurogenetics/GWAS-pipeline) using plink 1.9^48^. In brief, we filtered out heterozygosity outliers using an F-statistic cut-off of <−0.15 or >0.15. Individuals with a variant call rate <95% and sex mismatch were excluded. Variants missing in >5% of the participants, with disparate missingness between cases and controls (*p* < 1E−04), or significantly deviating from the Hardy-Weinberg equilibrium in controls (*p* < 1E−04) were also removed. We used GCTA to check for relatedness closer than first cousins between participants (pihat > 0.125). We performed imputation using the Michigan imputation server (https://imputationserver.sph.umich.edu/index.html#) with the Haplotype Reference Consortium reference panel r1.1 2016 under default settings. Ancestry outliers were detected using HapMap3 principal component analysis (PCA) data in R version 4.0.1. After imputation, we selected variants with *R*^2^ > 0.8 and a minor allele frequency (MAF) > 0.01, while retaining variants that have strong pathogenic implications in PD (i.e., the LRRK2 p.G2019S variant and the GBA1 p.N370S, p.E326K and p.T369M variants). After QC filtering, a total of 9,979,381 SNPs were analyzed in the GWAS for these cohorts.

### Statistical analysis

To test for genetic associations to RBD in PD, we performed GWAS using logistic regression comparing PD + RBD and PD-RBD adjusted for age at RBD questionnaire administration, sex and principal components. The significance threshold was set at *p* < 5 × 10E−8. The analyses were performed separately in each cohort and the results were then meta-analyzed with a fixed-effect model using METAL (https://genome.sph.umich.edu/wiki/METAL_Documentation)^49^. To identify any possible secondary associations hidden by the principal signals of the GWAS, we also performed Conditional and Joint–Genome-wide Complex Trait Analysis (COJO-GCTA), a method that harnesses a conditional stepwise regression approach to identify independent associations (https://yanglab.westlake.edu.cn/software/gcta/#Overview)^50^.

### Genetic correlation

To investigate the potential genetic correlation between the presence of RBD in PD and known neuropsychiatric conditions we used linkage-disequilibrium score regression (LDSC) on LDHub (http://ldsc.broadinstitute.org/ldhub/).^51^ The neuropsychiatric traits we analyzed include epilepsy, headache, amyotrophic lateral sclerosis, cognitive decline, Alzheimer’s disease, Parkinson’s disease, dementia with Lewy bodies, alcohol dependence, cannabis dependence, attention deficit hyperactivity disorder, Tourette syndrome, anorexia nervosa, post-traumatic syndrome, schizophrenia, bipolar disorder, obsessive-compulsive disorder, autism spectrum disorder and major depressive disorder. Summary statistics for the compared traits were accessed through the LDHub platform or downloaded from publicly available sources, then formatted and analyzed using LDHub python v2.7 scripts (https://github.com/bulik/ldsc/wiki/). Positive genetic correlations indicate positive association with genetic factors associated with RBD among individuals with PD.

### Mendelian randomization

To assess any possible causal association between neuropsychiatric disorders and the presence of RBD in PD we performed Mendelian randomization (MR)^52^. The neuropsychiatric traits for this analysis were selected based on their known clinical relevance to RBD or PD, as they have been reported in either RBD patients, PD patients or both. In brief, this method harnesses summary statistics from an exposure (the neuropsychiatric traits, in this case) and an outcome (the presence of RBD in PD) and uses the statistically significant variants from the former as instrumental variables (IVs) to infer a potential causative association with the latter. This approach mimics randomized control trials, since genetics is randomly assigned at conception and unaffected by the environment^53–56^. Differently from randomized control trials, however, MR relies on certain restrictive assumptions, varying based on the specific methods used, like the absence of horizontal pleiotropy and others, as well as on the quality of the GWASs they rely on. The neuropsychiatric traits for this analysis were selected based on relevance to RBD comorbidities, known neuropsychiatric manifestations in PD or with clinical relevance to PD. They include Alzheimer’s disease, dementia with Lewy Bodies, schizophrenia, major depressive disorder and bipolar disorder. We used the TwoSampleMR R package (https://mrcieu.github.io/TwoSampleMR/)^57^ to perform MR analyses, including sensitivity analyses, tests assessing pleiotropy and heterogeneity between IVs, in R version 4.0.1 according to protocols previously established^58^. Sensitivity analyses included MR Egger, inverse variance weighted (IVW), weighted median, simple mode and weighted mode. Steiger filtering was also performed to check for reverse causality. Summary statistics were downloaded by the MRBase GWAS catalog (http://www.mrbase.org/) and the Psychiatric Genomics Consortium (https://pgc.unc.edu/) publicly available database. To calculate the power to detect an odds ratio = 1.2 we used an online Mendelian Randomization power calculation tool (https://sb452.shinyapps.io/power/)^59^.

## Supplementary information

Supplementary materials

## Data Availability

The full PD with and without RBD GWAS summary statistics is detailed on the GWAS catalog (https://www.ebi.ac.uk/gwas/). The full GWAS summary statistics for the 23andMe dataset will be made available through 23andMe to qualified researchers under an agreement with 23andMe that protects the privacy of the 23andMe participants. Please visit https://research.23andme.com/collaborate/#dataset-access/ for more information and to apply to access the data. The GWAS summary statistics for the neuropsychiatric traits used in the study are available on the GWAS catalog and Psychiatric Genomics Consortium (https://pgc.unc.edu/).

## Code availability

The codes used for the analyses are available on https://github.com/daskrohn/RBD_GWAS and https://github.com/gan-orlab.
