# Supplementary material for: Genome-wide association study of REM sleep behavior disorder in Parkinson’s disease
Source: NPJ Parkinsons Dis. 2025 Sep 25;11:272. doi: 10.1038/s41531-025-01078-w (PMC12462436; doi:10.1038/s41531-025-01078-w)
Supplement: Supplementary file 1 — Supplementary materials [file 41531_2025_1078_MOESM1_ESM.pdf]

Genome-wide association study of REM sleep behavior disorder in Parkinson's disease

Supplementary Table 1 - PD GWAS hits in the PD with and without RBD GWAS compared with the previous PD GWAS in Europeans/Asians....5
Supplementary Table 2 - Carriers of the main *GBA1* pathogenic variants across different cohorts 6
Supplementary Table 3 - Genetic correlation between PD with RBD and neuropsychiatric traits....8
Supplementary Table 4: Two sample MR with neuropsychiatric traits as exposure and Parkinsons's disease with and without RBD as outcome....10
Supplementary Table 5 - Heterogeneity tests for two sample MR with Parkinsons's disease with and without RBD as outcome....11
Supplementary Table 6 - test for directional horizontal pleiotropy for two sample MR with Parkinsons's disease with and without RBD as outcome 12
Supplementary Table 7 - Test that the exposure is upstream of the outcome 13
Supplementary Figure 1 – Q-Q plot of the PD with and without RBD GWAS....14
Supplementary Figure 2 – Leave-one-out analyses in the Mendelian Randomization 20
Supplementary Figure 3 – Single variant analyses in the Mendelian Randomization 26
Comple list of authors, including IPDGC consortium and 23andMe members:....27
Affiliations....28

					PD with and without RBD		PD	
SNP	rs number	Reference allele	Alternative allele	Nearest gene	Beta	P-value	Beta	P-value
4:90636630	rs5019538	G	A	SNCA	-0.1644	2.46E-10	0.1565	1.13E-36
12:40734202	rs34637584	G	A	LRRK2	-0.8897	1.04E-05	2.4289	3.61E-148
4:90626111	rs356182	G	A	SNCA	-0.1147	1.37E-04	0.2774	3.89E-154
16:30977799	rs11150601	G	A	SETD1A	-0.0794	1.95E-03	0.0907	5.12E-20
19:2341047	rs55818311	C	T	SPPL2B	0.0857	2.51E-03	-0.0696	4.18E-10
17:43798308	rs117615688	G	A	CRHR1	0.1754	8.27E-03	-0.2324	6.71E-16
3:28705690	rs6808178	T	C	LINC00693	-0.0562	2.55E-02	0.0658	8.09E-12
11:133787001	rs3802920	G	T	IGSF9B	-0.0642	3.12E-02	0.1073	6.26E-20
2:169110394	rs1474055	C	T	STK39	0.0724	3.44E-02	0.1796	2.54E-39
12:133063768	rs11610045	G	A	FBRSL1	0.0603	3.79E-02	0.0601	1.77E-10
21:38852361	rs2248244	G	A	DYRK1A	-0.0569	3.72E-02	0.0714	2.74E-11
7:66009851	rs76949143	T	A	GS1-124K5.11	-0.1206	4.49E-02	-0.1432	1.43E-08
16:28944396	rs2904880	C	G	CD19	-0.0534	4.57E-02	-0.065	7.87E-10
1:155135036	rs35749011	G	A	KRTCAP2	0.1627	4.40E-02	0.6068	1.72E-70
2:18147848	rs76116224	A	T	KCNS3	-0.0851	4.64E-02	0.1104	1.27E-08
1:205723572	rs823118	C	T	NUCKS1	-0.0473	5.15E-02	0.1066	1.11E-29
7:23300049	rs199351	A	C	GPNMB	0.0467	6.02E-02	0.1016	5.25E-26
4:170583157	rs62333164	G	A	CLCN3	0.0479	6.63E-02	-0.0638	2.00E-10
5:134199105	rs11950533	C	A	C5orf24	-0.0748	7.22E-02	-0.0916	7.16E-09
14:88464264	rs979812	G	T	GALC	0.0432	7.59E-02	0.061	6.19E-11
13:49927732	rs9568188	T	C	CAB39L	-0.0459	9.55E-02	0.0617	1.15E-08

6:27738801	rs4140646	G	A	LOC100131289	-0.045	1.14E-01	0.0833	5.62E-12
10:121536327	rs117896735	G	A	INPP5F	0.1306	1.20E-01	0.4354	2.36E-28
17:42434630	rs850738	A	G	FAM171A2	-0.0377	1.32E-01	-0.071	1.29E-11
16:52636242	rs3104783	C	A	CASC16	-0.0366	1.38E-01	0.0668	1.29E-12
10:121415685	rs72840788	G	A	BAG3	0.042	1.47E-01	0.0763	1.57E-11
14:37989270	rs12147950	T	C	MIPOL1	0.0389	1.67E-01	-0.0529	3.54E-08
17:59917366	rs61169879	C	T	BRIP1	0.0445	1.74E-01	0.082	9.28E-10
14:55348869	rs11158026	C	T	GCH1	0.0345	1.85E-01	-0.0842	1.66E-16
17:7355621	rs12600861	A	C	CHRNB1	-0.0328	2.00E-01	-0.0565	1.01E-08
10:104015279	rs10748818	A	G	GBF1	0.0417	2.13E-01	-0.079	1.05E-09
1:232664611	rs10797576	C	T	SIPA1L2	-0.0414	2.40E-01	0.1114	6.84E-17
16:52969426	rs10221156	G	A	CHD9	-0.0495	2.44E-01	-0.1156	1.08E-10
20:6006041	rs77351827	C	T	CRLS1	0.0404	2.66E-01	0.0802	8.87E-09
3:161077630	rs1450522	A	G	SPTSSB	0.0284	2.70E-01	-0.0616	5.01E-10
6:72487762	rs12528068	C	T	RIMS1	-0.0301	2.63E-01	0.0657	1.63E-10
18:40673380	rs12456492	A	G	RIT2	0.0267	2.94E-01	-0.0983	3.80E-23
7:70750493	rs9638616	G	C	WBSCR17	-0.0257	3.26E-01	0.056904851	2.53E-08
3:48748989	rs12497850	G	T	IP6K2	0.0288	3.26E-01	0.0636	1.36E-10
1:155205634	rs76763715	T	C	GBAP1	-0.055	7.26E-01	-0.7467	1.59E-22
9:17727065	rs10756907	A	G	SH3GL2	0.0276	3.32E-01	-0.0926	5.06E-17
2:135464616	rs57891859	A	G	TMEM163	-0.0267	3.43E-01	0.0807	4.55E-14
1:171719769	rs11578699	C	T	VAMP4	0.0301	3.34E-01	-0.0704	4.47E-09
16:50736656	rs6500328	A	G	NOD2	-0.0345	3.67E-01	0.0586	1.82E-09
8:22525980	rs2280104	T	C	BIN3	0.0251	3.71E-01	0.0556	1.16E-08
1:226916078	rs4653767	T	C	ITPKB	-0.0234	3.91E-01	0.0833	1.38E-15

9:34046391	rs6476434	C	T	UBAP2	-0.0235	3.88E-01	-0.0615	6.58E-09
17:42294337	rs2269906	A	C	UBTF	0.0222	3.91E-01	0.0631	6.24E-10
12:40614434	rs76904798	C	T	LRRK2	0.0268	4.19E-01	0.1439	1.52E-28
4:114369065	rs13117519	C	T	CAMK2D	-0.0251	4.39E-01	0.0875	9.82E-13
17:76425480	rs666463	A	T	DNAH17	0.0257	4.34E-01	0.076	3.20E-09
14:75373034	rs3742785	A	C	RPS6KL1	-0.0228	4.47E-01	0.0707	1.92E-09
4:15737348	rs4698412	G	A	BST1	0.0183	4.49E-01	0.1035	2.06E-28
12:46419086	rs7134559	C	T	SCAF11	0.0186	4.59E-01	-0.0539	3.96E-08
3:182760073	rs10513789	T	G	MCCC1	-0.024	4.44E-01	0.1485	1.22E-34
1:205737739	rs11557080	G	A	RAB29	-0.0264	4.57E-01	0.1315	2.50E-22
5:75599208	rs246814	C	T	SV2C	-0.0299	4.91E-01	0.093421685	3.48E-08
6:133210361	rs75859381	T	C	RPS12	0.0488	4.98E-01	-0.2207	1.04E-10
6:32578772	rs112485576	C	A	HLA-DRB5	0.024	5.10E-01	-0.1676	6.96E-28
18:31304318	rs1941685	G	T	ASXL3	-0.0159	5.12E-01	0.0531	1.69E-08
4:951947	rs34311866	T	C	TMEM175	-0.0227	5.30E-01	-0.2126	9.98E-70
2:96000943	rs2042477	A	T	KCNIP3	-0.018	5.41E-01	-0.0657	1.38E-08
5:102365794	rs26431	G	C	PAM	-0.0143	5.95E-01	0.0621	1.57E-09
12:123326598	rs10847864	G	T	HIP1R	-0.0189	6.13E-01	0.1478	1.47E-37
4:77147969	rs4101061	A	G	FAM47E	-0.0138	6.01E-01	-0.0912	4.97E-19
17:43744203	rs62053943	C	T	CRHR1	0.0189	6.17E-01	-0.27	3.58E-68
17:40741013	rs12951632	T	C	RETREG3	0.0134	6.31E-01	0.0642	1.40E-09
5:60137959	rs1867598	A	G	ELOVL7	0.0195	6.25E-01	-0.1554	2.52E-23
6:112243291	rs997368	A	G	FYN	-0.0148	6.40E-01	0.0714	1.84E-09
10:15557406	rs896435	C	T	ITGA8	-0.0118	6.52E-01	0.0735	3.41E-13
1:161469054	rs6658353	G	C	FCGR2A	0.0105	6.67E-01	0.065	6.10E-12

3:151108965	rs11707416	T	A	MED12L	0.0112	6.58E-01	-0.0627	1.13E-10
3:122196892	rs55961674	C	T	KPNA1	0.0136	6.82E-01	0.0861	9.98E-12
4:17968811	rs34025766	T	A	LCORL	-0.0137	6.87E-01	-0.0839	2.87E-10
11:83487277	rs12283611	C	A	DLG2	-0.0081	7.45E-01	-0.0645	2.61E-10
17:44866805	rs11658976	G	A	WNT3	-0.0094	7.59E-01	-0.0624	3.52E-08
13:97865021	rs4771268	T	C	MBNL2	0.0085	7.69E-01	0.0675	1.45E-09
8:11712443	rs1293298	A	C	CTSB	-0.0089	7.58E-01	0.093	3.99E-16
4:77110365	rs6825004	C	G	SCARB2	-0.0066	8.01E-01	0.0622	1.17E-09
4:925376	rs873786	C	T	GAK	0.0107	7.99E-01	-0.1731	1.79E-21
2:102396963	rs11683001	T	A	MAP4K4	-0.0062	8.09E-01	0.0705	8.04E-13
16:19277493	rs6497339	A	T	SYT17	0.0073	7.90E-01	0.063	2.76E-11
3:18361759	rs73038319	A	C	SATB1	0.0154	7.99E-01	-0.1693	5.94E-13
15:61997385	rs2251086	T	C	VPS13C	0.0081	8.20E-01	-0.1186	6.08E-18
9:17579690	rs13294100	T	G	SH3GL2	-0.0044	8.66E-01	-0.0859	8.72E-18
4:77198054	rs6854006	C	T	FAM47E-STBD1	-0.0043	8.64E-01	-0.0912	5.82E-21
1:154898185	rs114138760	G	C	PMVK	0.0321	7.85E-01	0.2812	4.19E-09
8:16697593	rs620513	G	T	FGF20	0.0028	9.18E-01	-0.0856	2.72E-15
18:48683589	rs8087969	T	G	MEX3C	-0.0021	9.31E-01	-0.0578	1.41E-08
6:30108683	rs9261484	C	T	TRIM40	0.0012	9.65E-01	-0.0635	1.62E-08
11:10558777	rs7938782	A	G	RNF141	-0.0009	9.82E-01	0.087	2.12E-09
8:130901909	rs2086641	T	C	FAM49B	-0.0004	9.89E-01	-0.0605	1.81E-08

Supplementary Table 1 - PD GWAS hits in the PD with and without RBD GWAS compared with the previous PD GWAS in Europeans/Asians

PD: Parkinson's disease; RBD: REM sleep behavior disorder.

*hits statistically significant after Bonferroni correction (α=0.00054).

	Carriers PD-RBD (%)			Carriers PD+RBD (%)		
**Cohort**	**p.N370S**	**p.T369M**	**p.E326K**	**p.N370S**	**p.T369M**	**p.E326K**
**Oslo**	0.01%	0.56%	0.05%	0.00%	0.02%	0.05%
**Lund**	0.18%	2.34%	6.85%	1.64%	2.47%	6.03%
**McGill**	0.01%	0.37%	0.37%	0.01%	0.21%	0.39%
**AMP-PD**	0.03%	0.02%	0.03%	0.02%	0.01%	0.06%
**Sydney**	0.71%	0.01%	0.06%	0.00%	0.00%	0.02%
**Tuebingen**	2.34%	3.43%	5.77%	2.19%	2.46%	6.30%
**Barcelona**	0.00%	0.00%	0.02%	0.01%	0.01%	0.00%
**PRoBaND**	0.53%	1.50%	3.97%	0.34%	1.71%	6.67%
**PFP**	3.56%	1.49%	0.88%	4.69%	1.58%	0.00%
**OPDC**	0.19%	1.67%	5.81%	1.46%	2.19%	2.92%
**Kosice**	0.00%	0.03%	0.03%	0.00%	0.04%	0.02%
**23andMe**	1.77%	2.15%	3.14%	1.84%	1.41%	3.67%
**TOTAL**	0.78%	1.13%	2.25%	1.02%	1.01%	2.18%

Supplementary Table 2 - Carriers of the main *GBA1* pathogenic variants across different cohorts

PD-RBD: participants without REM sleep behavior disorder (RBD); PD+RBD: participants with RBD; Oslo: Oslo University Hospital; Lund: Lund University; McGill: McGill University;

AMP-PD: Accelerating Medicines Partnership Parkinson's disease, including the New Discovery of Biomarkers (BioFIND), the Harvard Biomarker Study (HBS) and the Parkinson's Disease Biomarkers Program (PDBP) cohorts;

Sydney: University of Sydney; Tuebingen: University of Tuebingen; Barcelona: Hospital Universitari Mutua de Terrassa; PRoBaND: Parkinson's repository of biosamples and networked datasets;

PFP: Parkinson's Families Project; OPDC: Oxford Parkinson's Disease Centre; Kosice: Pavol Jozef Šafárik University in Kosice; TOTAL: average percentage of carriers across cohorts.

trait1	trait2	rg	se	z	p	h2_obs	h2_obs_se	h2_int	h2_int_se	gcov_int	gcov_int_se
PDw RBD	HEADACHE	0.1002	0.099	1.0124	0.3113	0.0872	0.0042	1.0181	0.0089	-0.0123	0.0053
PDw RBD	EPILEPSY	0.2936	0.2013	1.4584	0.1447	0.1072	0.0162	1.1674	0.0112	-0.0139	0.0065
PDw RBD	ALS	0.0452	0.2051	0.2203	0.8256	0.0296	0.007	1.023	0.0073	0.007	0.0043
PDw RBD	CD	0.1064	0.1981	0.5372	0.5911	0.168	0.0074	1.0246	0.0219	-0.02	0.0115
PDw RBD	AD	0.1301	0.1974	0.6591	0.5098	0.0076	0.0048	1.0559	0.0583	0.0063	0.0051
PDw RBD	PD	-0.3813	0.1534	-2.4853	0.0129	0.0193	0.002	0.9778	0.007	0.0141	0.0043
PDw RBD	DLB	0.4435	0.4481	0.9899	0.3222	0.1407	0.0748	1.0044	0.0087	-0.0018	0.0046
PDw RBD	ALCOL DEP	0.0817	0.2339	0.3493	0.7268	50.3589	9.451	1.0196	0.0062	0.0032	0.0043
PDw RBD	CANNABIS DEP	-0.0744	0.1131	-0.6573	0.511	0.0675	0.0043	1.0015	0.0076	0.0078	0.0051
PDw RBD	ADHD	0.2953	0.1442	2.0481	0.0406	0.2402	0.0151	1.0297	0.0101	-0.0029	0.0056
PDw RBD	OCD	0.0129	0.1807	0.0715	0.943	0.3272	0.0483	0.9917	0.0065	0.0018	0.0044
PDw RBD	ASD	0.1341	0.138	0.9716	0.3313	0.2016	0.0147	1.0005	0.008	-0.0034	0.0051
PDw RBD	TS	0.0316	0.1632	0.1938	0.8464	0.3532	0.0415	1.0167	0.0073	0.0066	0.0046
PDw RBD	AN	0.1113	0.1312	0.8483	0.3963	0.1791	0.0114	1.0238	0.0099	-0.0186	0.0052
PDw RBD	PTS	-0.0986	0.2003	-0.4923	0.6225	0.017	0.0029	1.0208	0.0066	0.0191	0.0043
PDw RBD	SCZ	0.0148	0.0783	0.1885	0.8505	0.3379	0.0108	1.0602	0.0136	0.007	0.0059
PDw RBD	BD	0.1075	0.1029	1.0449	0.2961	0.3447	0.0159	1.0236	0.008	0.003	0.0051
PDw RBD	MDD	0.092	0.1248	0.7371	0.4611	0.0727	0.0043	0.9968	0.0073	0.0037	0.0046

Supplementary Table 3 - Genetic correlation between PD with RBD and neuropsychiatric traits

rg = genetic correlation; se = standard error of rg; p = p-value for rg; z= z-score; h2_obs, h2_obs_se = observed scale heritability for trait 2 and standard error

h2_int, h2_int_se = single-trait LD Score regression intercept for trait 2 and standard error; gcov_int, gcov_int_se = cross-trait LD Score regression intercept and standard error

ALS: amyotrophic lateral sclerosis; CD: cognitive decline; AD: Alzheimer's disease; PD: Parkinson's disease; DLB: dementia with Lewy bodies;

Alcol dep: alcol dependence; cannabis dep: cannabis dependence; ADHD: attention deficit hyperactivity disorder; TS: Tourette syndrome;

AN: anorexia nervosa; PTS: post-traumatic syndrome; scz: schizophrenia; BD: bipolar disorder; MDD: major depressive disorder

exposure	method	nsnp	b	se	pval
AD	MR Egger	19	-0.264946	0.352434	0.462476
	Weighted median	19	0.134947	0.338085	0.689782
	Inverse variance weighted	19	-0.110972	0.240938	0.645097
	Simple mode	19	0.441533	0.549944	0.432525
	Weighted mode	19	0.175078	0.363578	0.635934
DLB	MR Egger	5	0.025903	0.20084	0.905541
	Weighted median	5	0.06753	0.037263	0.069944
	Inverse variance weighted	5	0.133277	0.111793	0.233195
	Simple mode	5	0.09336	0.055787	0.169542
	Weighted mode	5	0.067159	0.040258	0.170595
SCZ	MR Egger	191	0.243161	0.132616	0.06829
	Weighted median	191	0.01571	0.049733	0.752091
	Inverse variance weighted	191	0.026	0.034227	0.447472
	Simple mode	191	0.285014	0.163496	0.082909
	Weighted mode	191	0.290541	0.194105	0.1361
Depr	MR Egger	31	0.970045	1.049482	0.362954
	Weighted median	31	0.099923	0.220759	0.650812
	Inverse variance weighted	31	0.084974	0.161921	0.599731
	Simple mode	31	0.50272	0.439164	0.261376
	Weighted mode	31	0.54446	0.425088	0.210072
Bip	MR Egger	53	0.536276	0.295617	0.075549
	Weighted median	53	0.068324	0.083532	0.413393
	Inverse variance weighted	53	0.036292	0.057231	0.525997

	Simple mode	53	0.08223	0.223349	0.714243
	Weighted mode	53	0.101594	0.227871	0.657562

Supplementary Table 4: Two sample MR with neuropsychiatric traits as exposure and Parkinsons's disease with and without RBD as outcome

MR: mendelian randomization; nsnp: number of snps included; b: beta coefficient; se: standard error; pval: p-value;

RBD: REM sleep behavior disorder; AD: Alzheimer's disease; DLB: Dementia with Lewy Bodies; SCZ: schizophrenia; Depr: depression; Bip: Bipolar disorder

exposure	method	Q	Q_df	Q_pval
AD	MR Egger	4.494828	17	0.998877
	Inverse variance weighted	4.85318	18	0.99907
SCZ	MR Egger	150.4621	189	0.982166
	Inverse variance weighted	153.3349	190	0.976307
DLB	MR Egger	42.1884	3	0
	Inverse variance weighted	48.46504	4	0
Depr	MR Egger	12.72608	29	0.996165
	Inverse variance weighted	13.45464	30	0.99597
Bip	MR Egger	45.75078	51	0.681566
	Inverse variance weighted	48.72274	52	0.603568

Supplementary Table 5 - Heterogeneity tests for two sample MR with Parkinsons's disease with and without RBD as outcome

MR: mendelian randomization; AD: Alzheimer's disease; DLB: Dementia with Lewy Bodies;

SCZ: schizophrenia; Depr: depression; Bip: Bipolar disorder

exposure	egger_intercept	se	pval
AD	0.0064164	0.010719	0.557316
SCZ	-0.0133813	0.007895	0.091733
DLB	0.0553819	0.082897	0.551887
Depr	-0.0270587	0.031701	0.400344
Bip	-0.0340643	0.01976	0.090777

Supplementary Table 6 - test for directional horizontal pleiotropy for two sample MR with Parkinsons's disease with and without RBD as outcome

MR: mendelian randomization; AD: Alzheimer's disease; DLB: Dementia with Lewy Bodies; SCZ: schizophrenia; Depr: depression; Bip: Bipolar disorder

exposure	snp_r2.exposure	snp_r2.outcome	correct_causal_direction	steiger_pval
AD	0	0		0
DLB	0	0		0
SCZ	0	0		0
Depr	0	0		0
Bip	0	0		0

Supplementary Table 7 - Test that the exposure is upstream of the outcome

AD: Alzheimer's disease; DLB: Dementia with Lewy Bodies; SCZ: schizophrenia; Depr: depression; Bip: Bipolar disorder

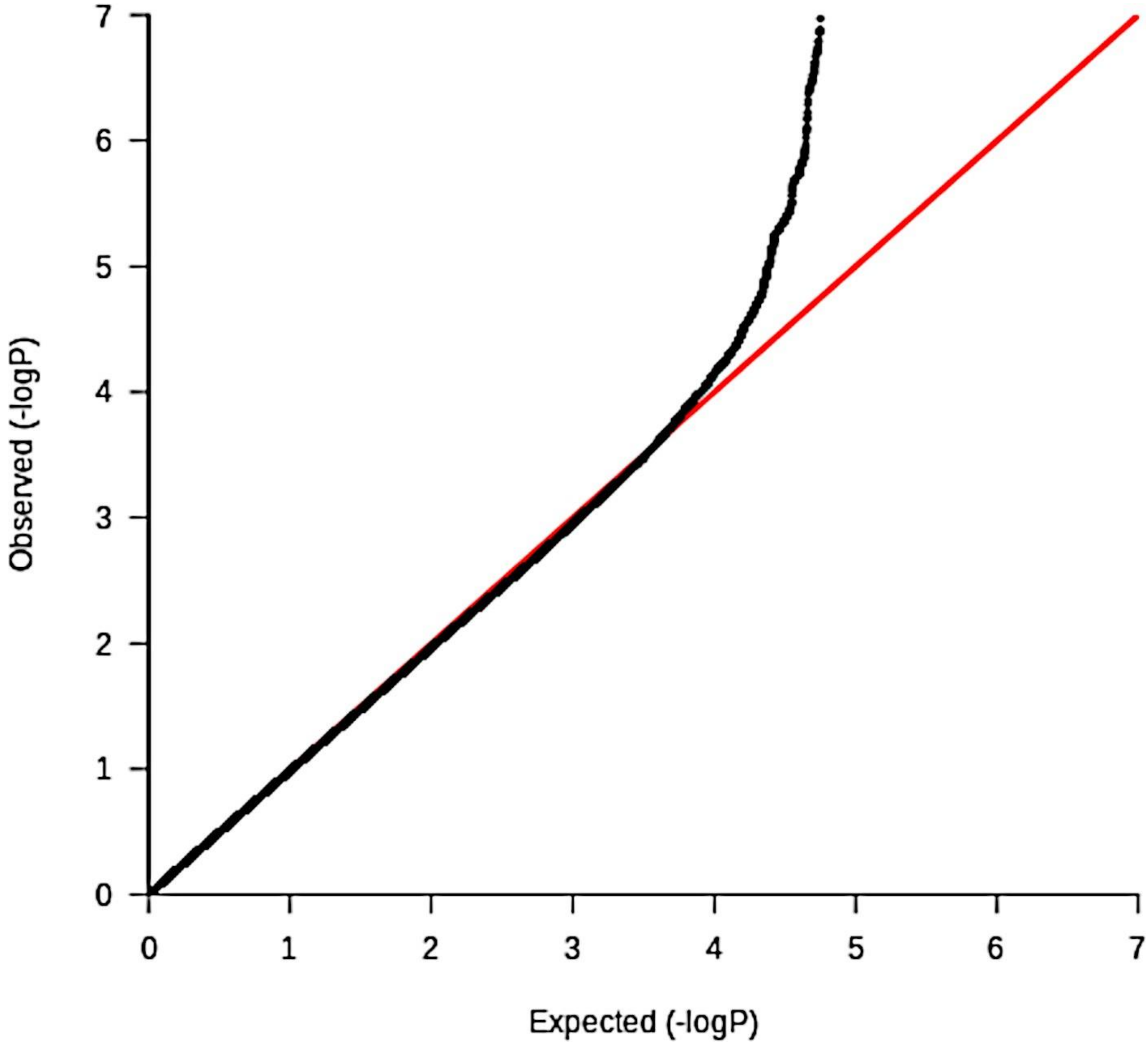

Supplementary Figure 1 – Q-Q plot of the PD with and without RBD GWAS

The Q-Q plot illustrates the negative log-adjusted p-values from the GWAS sorted into ascending order against the expected quantiles if the null hypothesis is true for all tests.

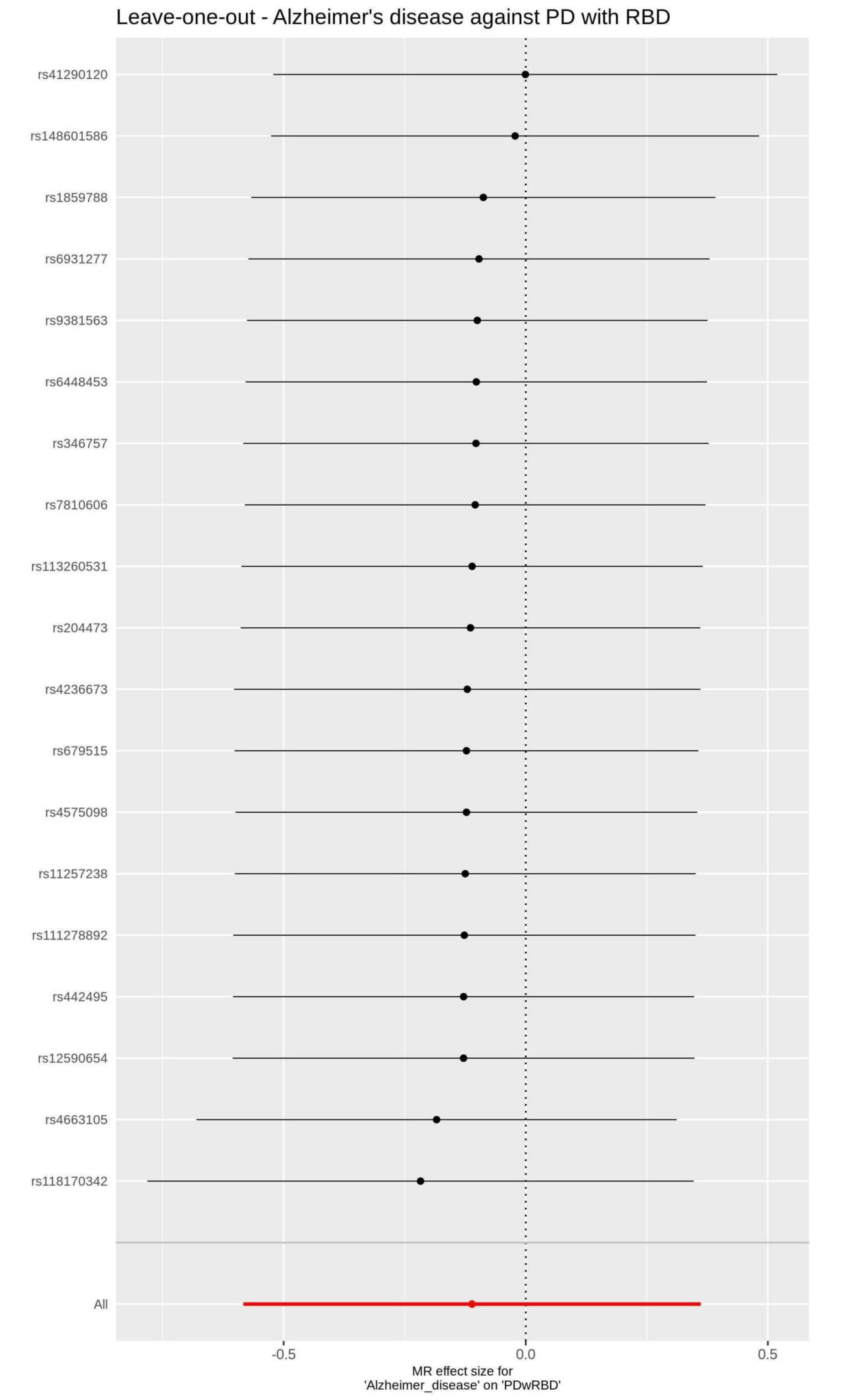

Leave-one-out - Alzheimer's disease against PD with RBD
rs41290120
rs148601586
rs1859788
rs6931277
rs9381563
rs6448453
rs346757
rs7810606
rs113260531
rs204473
rs4236673
rs679515
rs4575098
rs11257238
rs111278892
rs442495
rs12590654
rs4663105
rs118170342
All
-0.5
0.0
0.5
MR effect size for
'Alzheimer_disease' on 'PDwRBD'

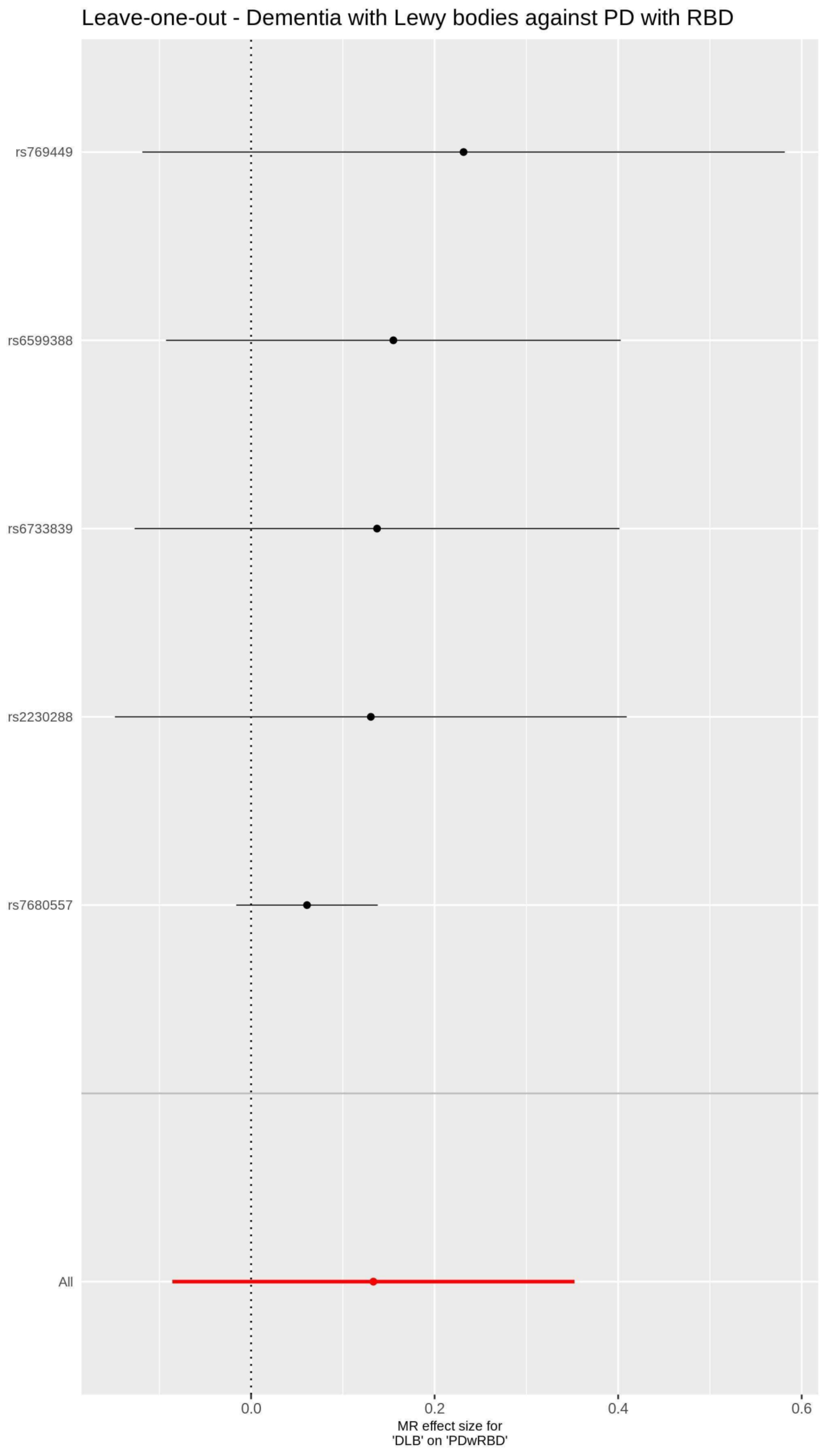

Leave-one-out - Dementia with Lewy bodies against PD with RBD
rs769449
rs6599388
rs6733839
rs2230288
rs7680557
All
0.0
0.2
0.4
0.6
MR effect size for
'DLB' on 'PDwRBD'

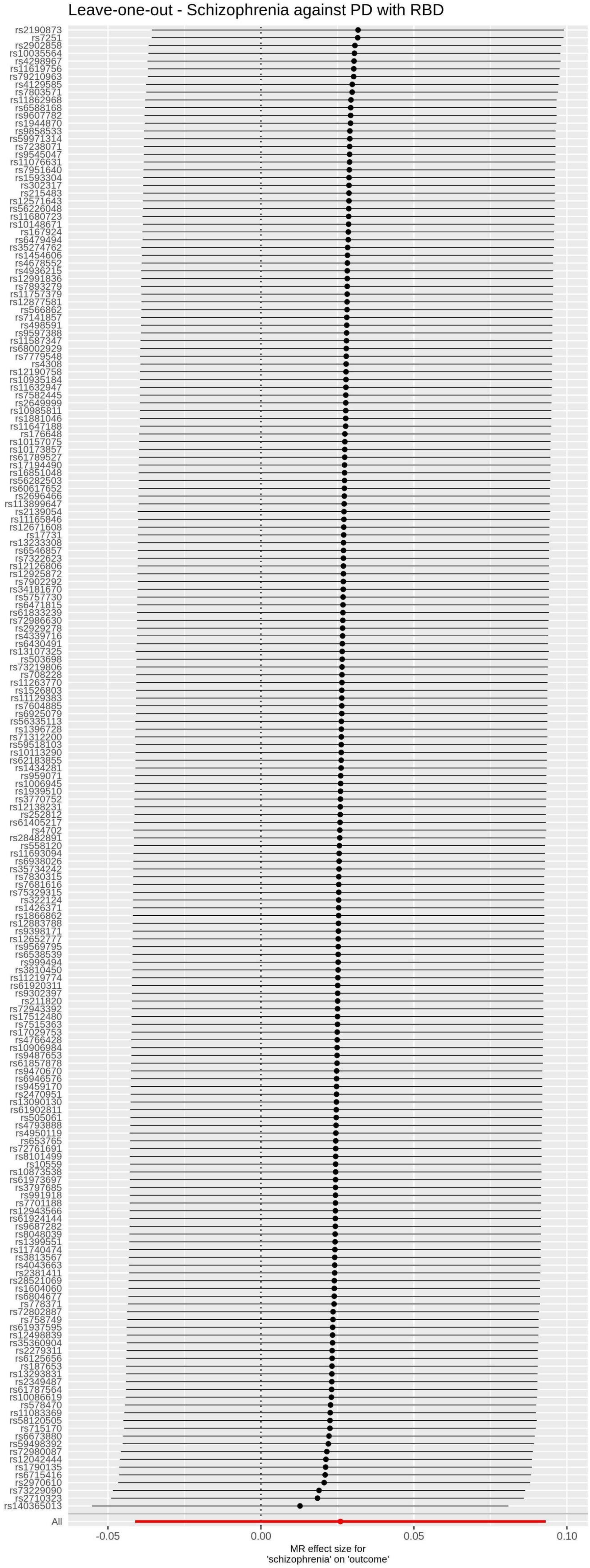
Leave-one-out - Schizophrenia against PD with RBD
rs2190873
rs7251
rs2902858
rs10035564
rs4298967
rs11619756
rs79210963
rs4129585
rs7803571
rs11862968
rs6588168
rs9607782
rs1944870
rs9858533
rs59971314
rs7238071
rs9545047
rs11076631
rs7951640
rs1593304
rs302317
rs215483
rs12571643
rs56226048
rs11680723
rs10148671
rs167924
rs6479494
rs35274762
rs1454606
rs4678552
rs4936215
rs12991836
rs7893279
rs11757379
rs12877581
rs566862
rs7141857
rs498591
rs9597388
rs11587347
rs68002929
rs7779548
rs4308
rs12190758
rs10935184
rs11632947
rs7582445
rs2649999
rs10985811
rs1881046
rs11647188
rs176648
rs10157075
rs10173857
rs61789527
rs17194490
rs16851048
rs56282503
rs60617652
rs2696466
rs113899647
rs2139054
rs11165846
rs12671608
rs17731
rs13233308
rs6546857
rs7322623
rs12126806
rs12925872
rs7902292
rs34181670
rs5757730
rs6471815
rs61833239
rs72986630
rs2929278
rs4339716
rs6430491
rs13107325
rs503698
rs73219806
rs708228
rs11263770
rs1526803
rs11129383
rs7604885
rs6925079
rs56335113
rs1396728
rs71312200
rs59518103
rs10113290
rs62183855
rs1434281
rs959071
rs1006945
rs1939510
rs3770752
rs12138231
rs252812
rs61405217
rs4702
rs28482891
rs558120
rs11693094
rs6938026
rs35734242
rs7830315
rs7681616
rs75329315
rs322124
rs1426371
rs1866862
rs12883788
rs9398171
rs12652777
rs9569795
rs6538539
rs999494
rs3810450
rs11219774
rs61920311
rs9302397
rs211820
rs72943392
rs17512480
rs7515363
rs17029753
rs4766428
rs10906984
rs9487653
rs61857878
rs9470670
rs6946576
rs9459170
rs2470951
rs13090130
rs61902811
rs505061
rs4793888
rs4950119
rs653765
rs72761691
rs8101499
rs10559
rs10873538
rs61973697
rs3797685
rs991918
rs7701188
rs12943566
rs61924144
rs9687282
rs8048039
rs1399551
rs11740474
rs3813567
rs4043663
rs2381411
rs28521069
rs1604060
rs6804677
rs778371
rs72802887
rs758749
rs61937595
rs12498839
rs35360904
rs2279311
rs6125656
rs187653
rs13293831
rs2349487
rs61787564
rs10086619
rs578470
rs11083369
rs58120505
rs715170
rs6673880
rs59498392
rs72980087
rs12042444
rs1790135
rs6715416
rs2970610
rs73229090
rs2710323
rs140365013
All
-0.05
0.00
0.05
0.10
MR effect size for
'schizophrenia' on 'outcome'

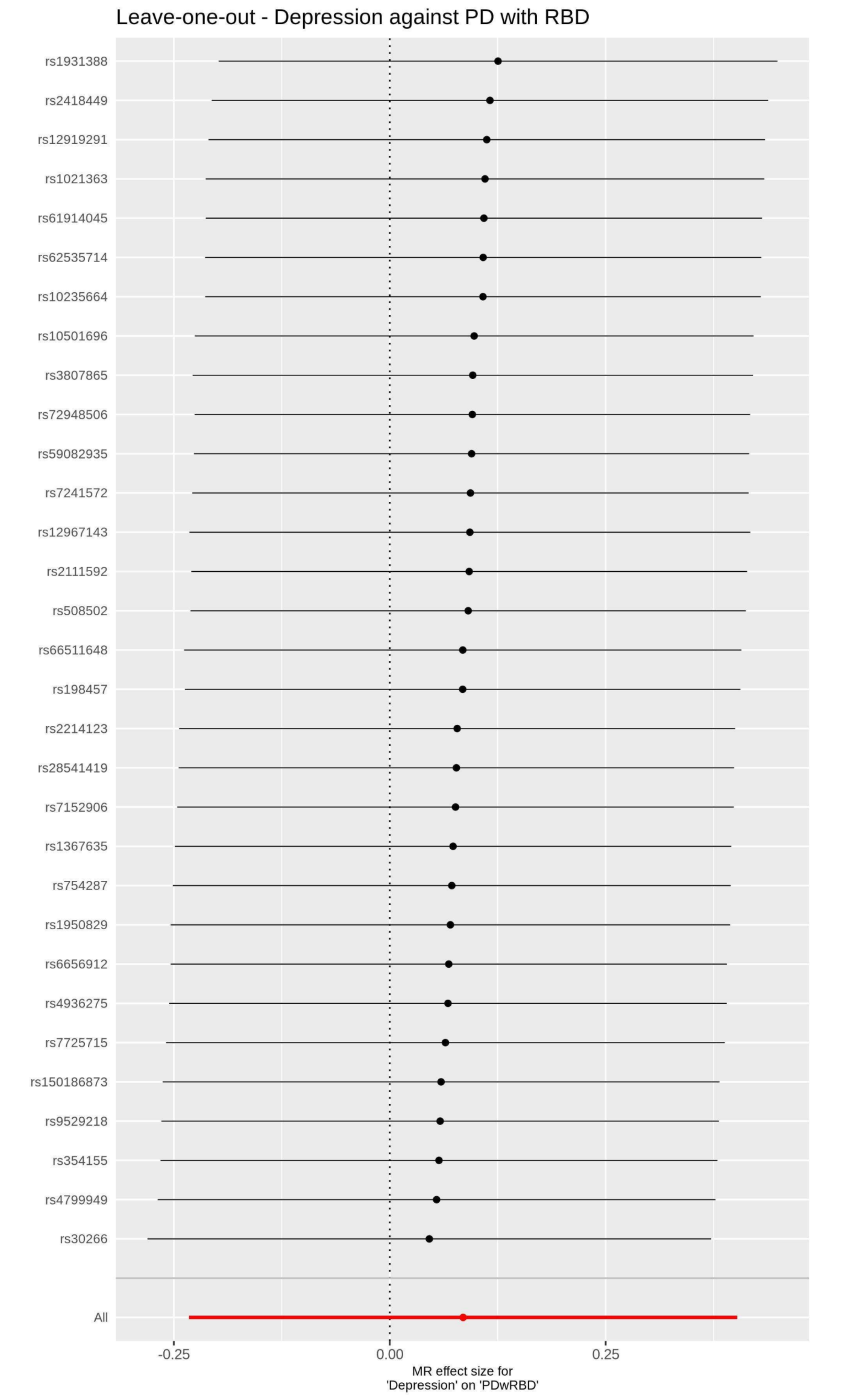

Leave-one-out - Depression against PD with RBD
rs1931388
rs2418449
rs12919291
rs1021363
rs61914045
rs62535714
rs10235664
rs10501696
rs3807865
rs72948506
rs59082935
rs7241572
rs12967143
rs2111592
rs508502
rs66511648
rs198457
rs2214123
rs28541419
rs7152906
rs1367635
rs754287
rs1950829
rs6656912
rs4936275
rs7725715
rs150186873
rs9529218
rs354155
rs4799949
rs30266
All
-0.25
0.00
0.25
MR effect size for
'Depression' on 'PDwRBD'

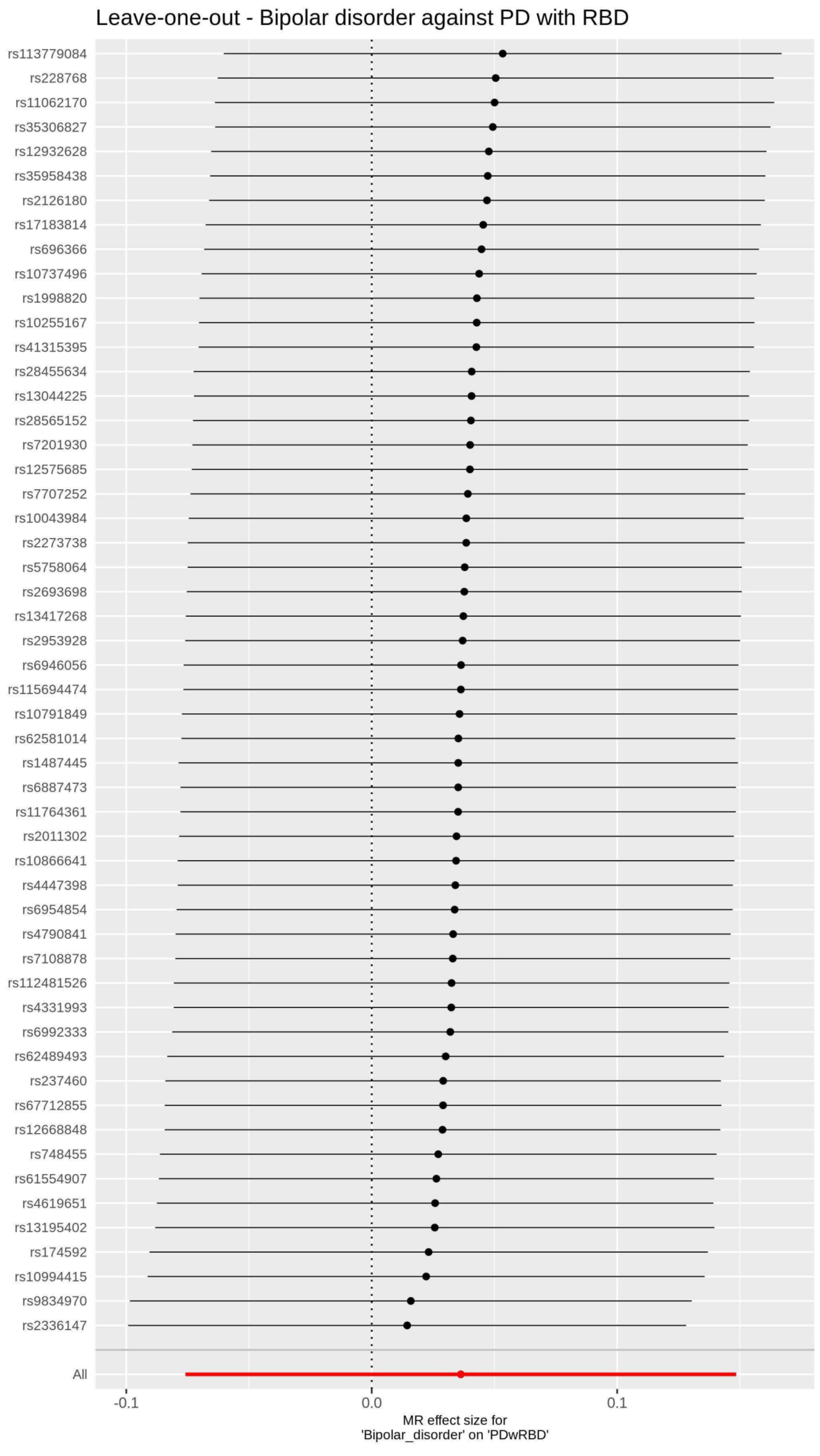

Leave-one-out - Bipolar disorder against PD with RBD
rs113779084
rs228768
rs11062170
rs35306827
rs12932628
rs35958438
rs2126180
rs17183814
rs696366
rs10737496
rs1998820
rs10255167
rs41315395
rs28455634
rs13044225
rs28565152
rs7201930
rs12575685
rs7707252
rs10043984
rs2273738
rs5758064
rs2693698
rs13417268
rs2953928
rs6946056
rs115694474
rs10791849
rs62581014
rs1487445
rs6887473
rs11764361
rs2011302
rs10866641
rs4447398
rs6954854
rs4790841
rs7108878
rs112481526
rs4331993
rs6992333
rs62489493
rs237460
rs67712855
rs12668848
rs748455
rs61554907
rs4619651
rs13195402
rs174592
rs10994415
rs9834970
rs2336147
All
-0.1
0.0
0.1
MR effect size for
'Bipolar_disorder' on 'PDwRBD'

Supplementary Figure 2 – Leave-one-out analyses in the Mendelian Randomization

The plots show the Mendelian Randomization coefficient (black dot) and 95% confidence interval (bars) when each variant is excluded from the analysis. The excluded variant is indicated on the Y axis. The red dot and line represent the results if all the variants are included.

SNP: single nucleotide polymorphism. MR: Mendelian Randomization; PD: Parkinson’s disease; RBD: REM sleep behavior disorder.

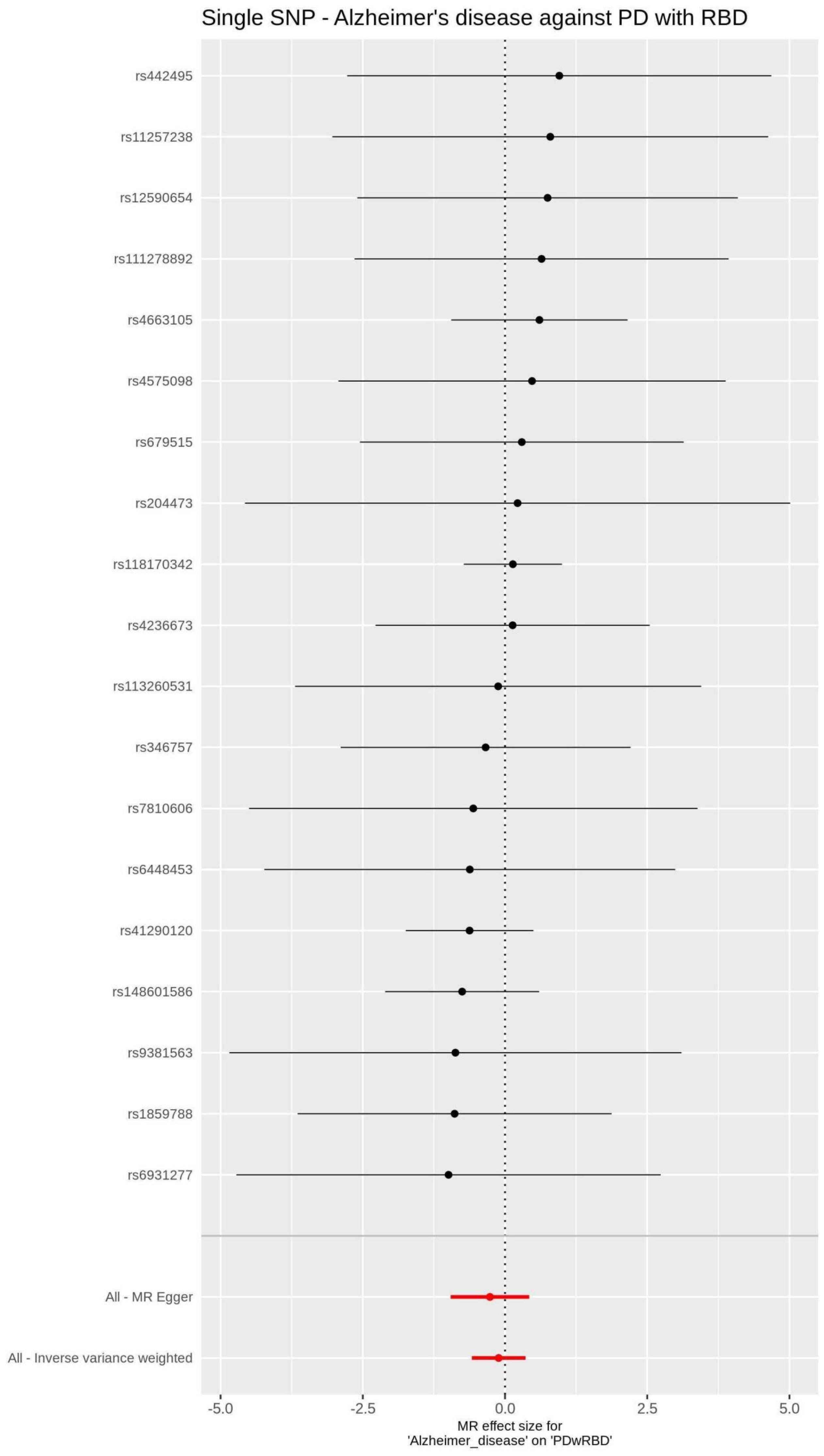
Single SNP - Alzheimer's disease against PD with RBD
rs442495
rs11257238
rs12590654
rs111278892
rs4663105
rs4575098
rs679515
rs204473
rs118170342
rs4236673
rs113260531
rs346757
rs7810606
rs6448453
rs41290120
rs148601586
rs9381563
rs1859788
rs6931277
All - MR Egger
All - Inverse variance weighted
-5.0
-2.5
0.0
2.5
5.0
MR effect size for
'Alzheimer_disease' on 'PDwRBD'

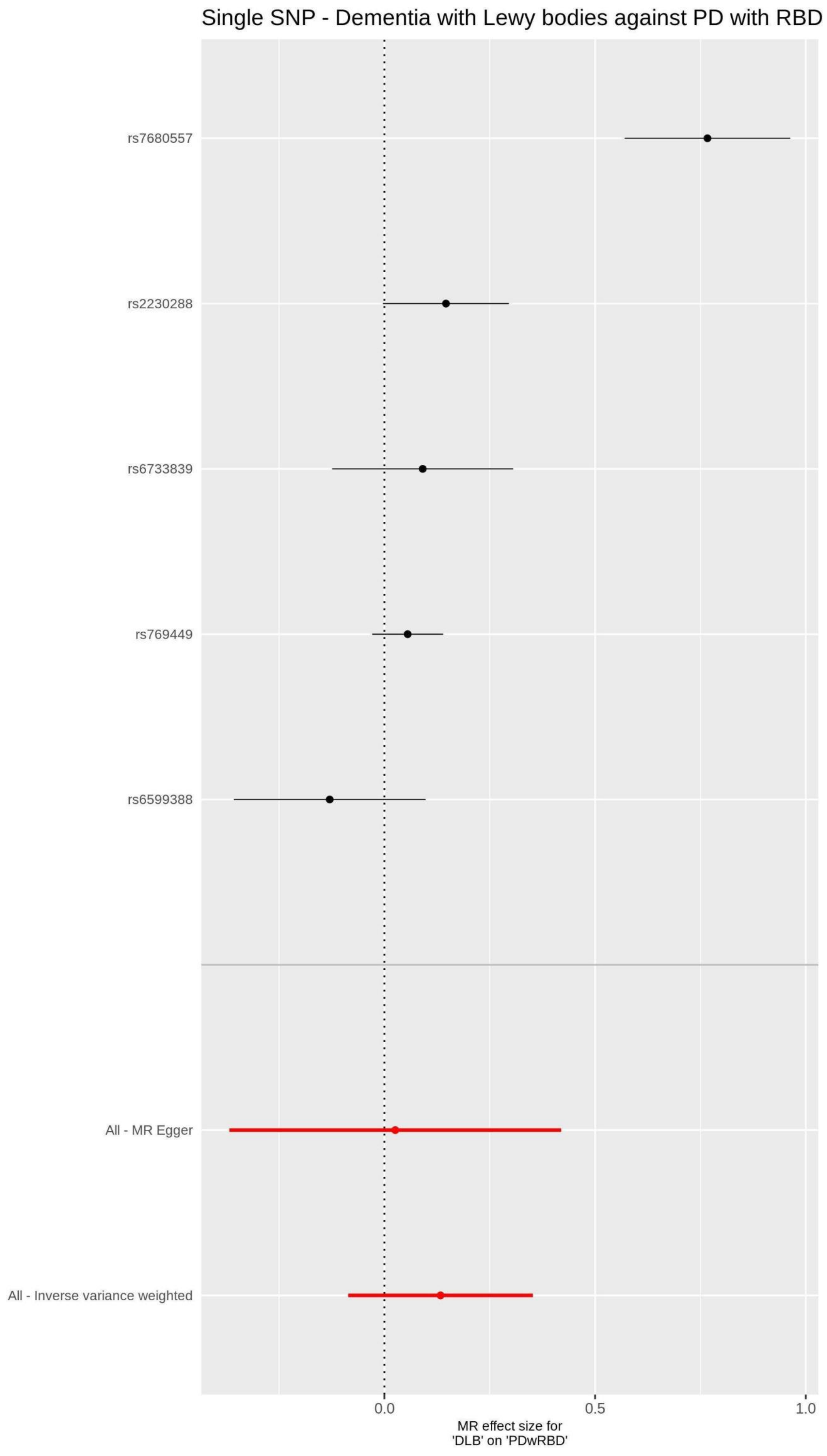

Single SNP - Dementia with Lewy bodies against PD with RBD
rs7680557
rs2230288
rs6733839
rs769449
rs6599388
All - MR Egger
All - Inverse variance weighted
0.0
0.5
1.0
MR effect size for
'DLB' on 'PDwRBD'

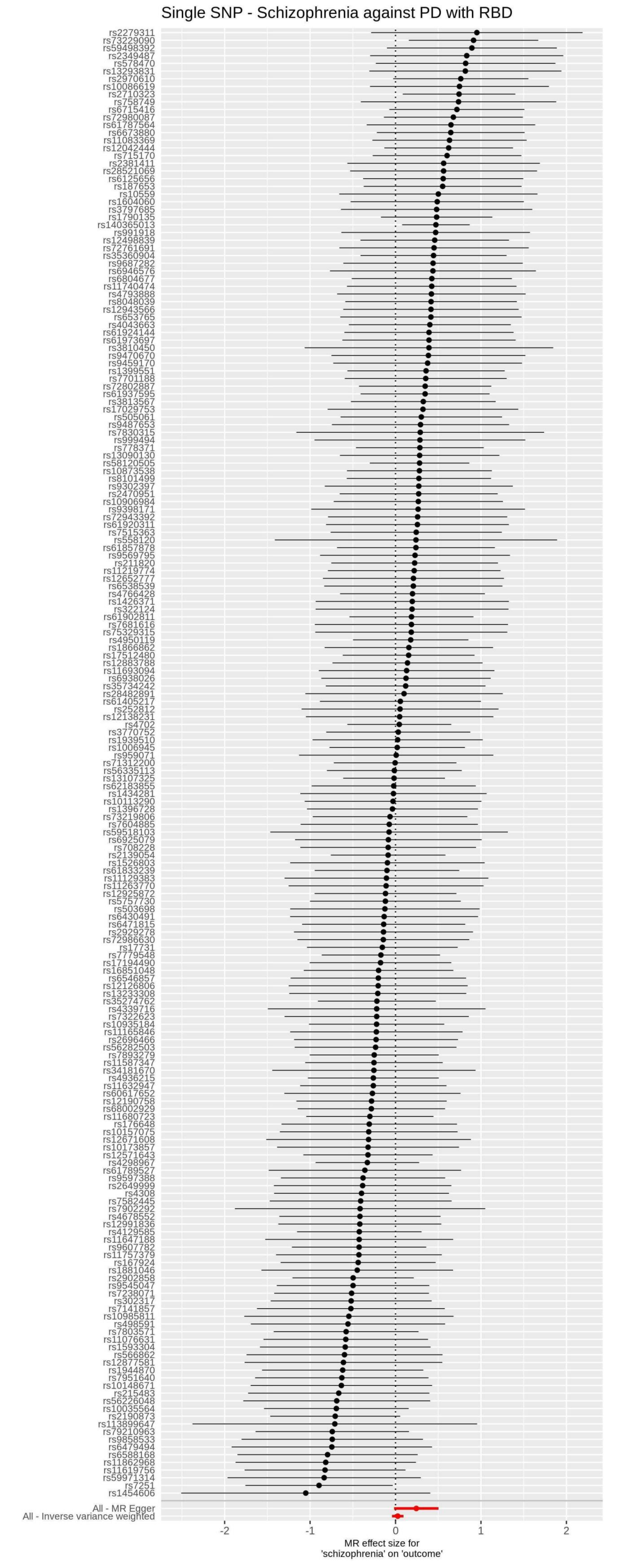
Single SNP - Schizophrenia against PD with RBD
rs2279311
rs73229090
rs59498392
rs2349487
rs578470
rs13293831
rs2970610
rs10086619
rs2710323
rs758749
rs6715416
rs72980087
rs61787564
rs6673880
rs11083369
rs12042444
rs715170
rs2381411
rs28521069
rs6125656
rs187653
rs10559
rs1604060
rs3797685
rs1790135
rs140365013
rs991918
rs12498839
rs72761691
rs35360904
rs9687282
rs6946576
rs6804677
rs11740474
rs4793888
rs8048039
rs12943566
rs653765
rs4043663
rs61924144
rs61973697
rs3810450
rs9470670
rs9459170
rs1399551
rs7701188
rs72802887
rs61937595
rs3813567
rs17029753
rs505061
rs9487653
rs7830315
rs999494
rs778371
rs13090130
rs58120505
rs10873538
rs8101499
rs9302397
rs2470951
rs10906984
rs9398171
rs72943392
rs61920311
rs7515363
rs558120
rs61857878
rs9569795
rs211820
rs11219774
rs12652777
rs6538539
rs4766428
rs1426371
rs322124
rs61902811
rs7681616
rs75329315
rs4950119
rs1866862
rs17512480
rs12883788
rs11693094
rs6938026
rs35734242
rs28482891
rs61405217
rs252812
rs12138231
rs4702
rs3770752
rs1939510
rs1006945
rs959071
rs71312200
rs56335113
rs13107325
rs62183855
rs1434281
rs10113290
rs1396728
rs73219806
rs7604885
rs59518103
rs6925079
rs708228
rs2139054
rs1526803
rs61833239
rs11129383
rs11263770
rs12925872
rs5757730
rs503698
rs6430491
rs6471815
rs2929278
rs72986630
rs17731
rs7779548
rs17194490
rs16851048
rs6546857
rs12126806
rs13233308
rs35274762
rs4339716
rs7322623
rs10935184
rs11165846
rs2696466
rs56282503
rs7893279
rs11587347
rs34181670
rs4936215
rs11632947
rs60617652
rs12190758
rs68002929
rs11680723
rs176648
rs10157075
rs12671608
rs10173857
rs12571643
rs4298967
rs61789527
rs9597388
rs2649999
rs4308
rs7582445
rs7902292
rs4678552
rs12991836
rs4129585
rs11647188
rs9607782
rs11757379
rs167924
rs1881046
rs2902858
rs9545047
rs7238071
rs302317
rs7141857
rs10985811
rs498591
rs7803571
rs11076631
rs1593304
rs566862
rs12877581
rs1944870
rs7951640
rs10148671
rs215483
rs56226048
rs10035564
rs2190873
rs113899647
rs79210963
rs9858533
rs6479494
rs6588168
rs11862968
rs11619756
rs59971314
rs7251
rs1454606
All - MR Egger
All - Inverse variance weighted
-2
-1
0
1
2
MR effect size for
'schizophrenia' on 'outcome'

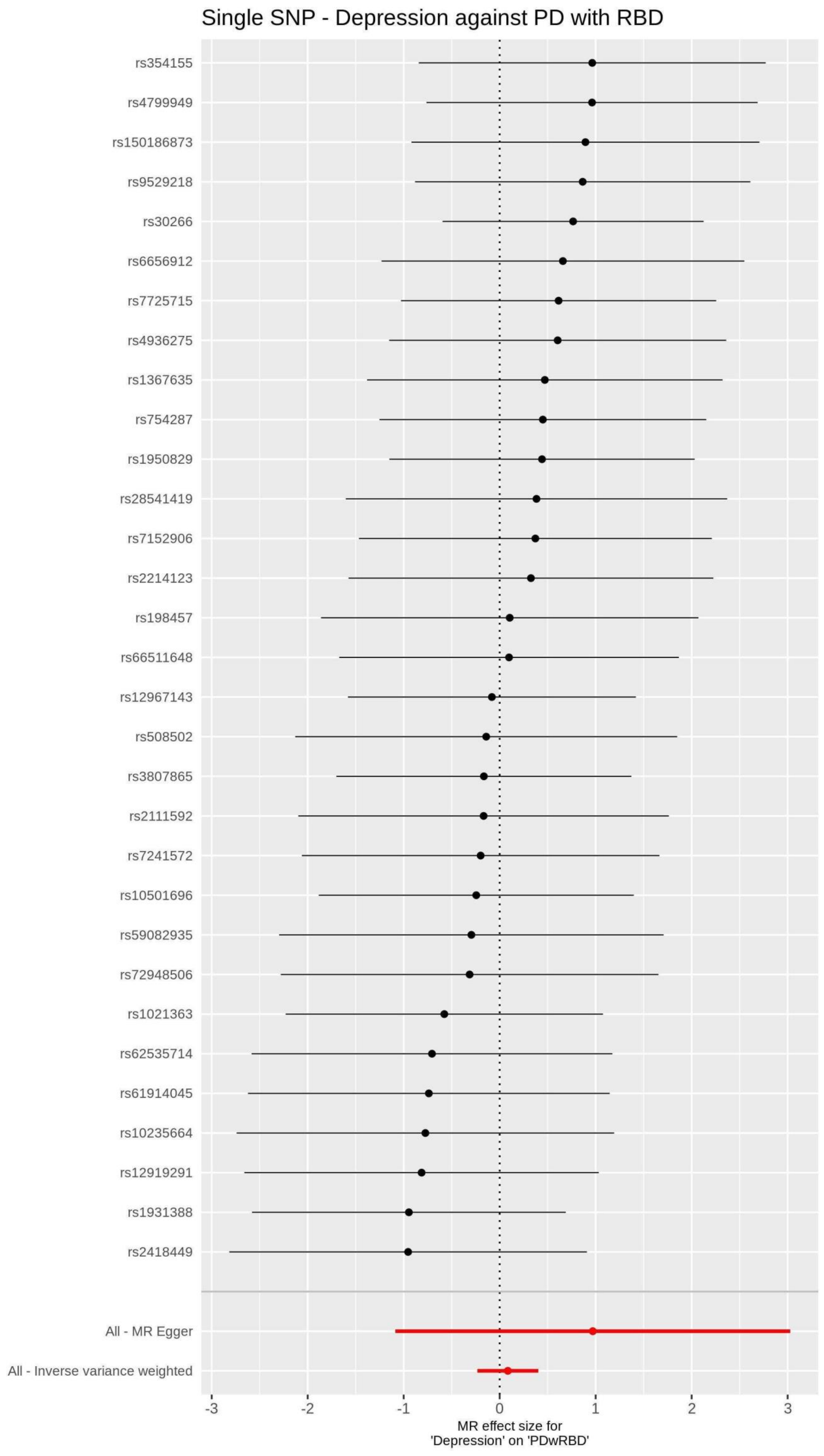
Single SNP - Depression against PD with RBD
rs354155
rs4799949
rs150186873
rs9529218
rs30266
rs6656912
rs7725715
rs4936275
rs1367635
rs754287
rs1950829
rs28541419
rs7152906
rs2214123
rs198457
rs66511648
rs12967143
rs508502
rs3807865
rs2111592
rs7241572
rs10501696
rs59082935
rs72948506
rs1021363
rs62535714
rs61914045
rs10235664
rs12919291
rs1931388
rs2418449
All - MR Egger
All - Inverse variance weighted
-3
-2
-1
0
1
2
3
MR effect size for
'Depression' on 'PDwRBD'

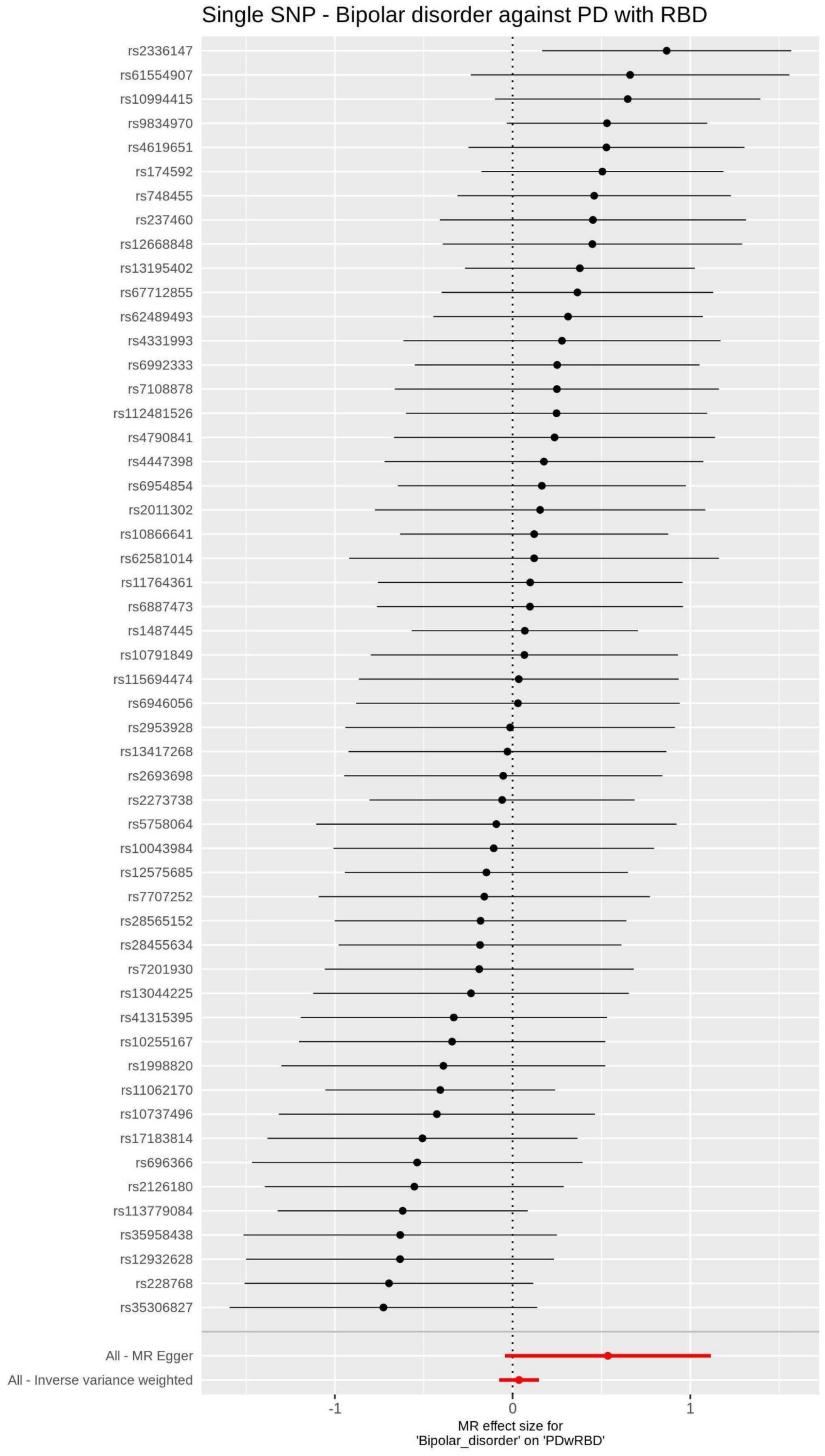
Single SNP - Bipolar disorder against PD with RBD
rs2336147
rs61554907
rs10994415
rs9834970
rs4619651
rs174592
rs748455
rs237460
rs12668848
rs13195402
rs67712855
rs62489493
rs4331993
rs6992333
rs7108878
rs112481526
rs4790841
rs4447398
rs6954854
rs2011302
rs10866641
rs62581014
rs11764361
rs6887473
rs1487445
rs10791849
rs115694474
rs6946056
rs2953928
rs13417268
rs2693698
rs2273738
rs5758064
rs10043984
rs12575685
rs7707252
rs28565152
rs28455634
rs7201930
rs13044225
rs41315395
rs10255167
rs1998820
rs11062170
rs10737496
rs17183814
rs696366
rs2126180
rs113779084
rs35958438
rs12932628
rs228768
rs35306827
All - MR Egger
All - Inverse variance weighted
-1
0
1
MR effect size for
'Bipolar_disorder' on 'PDwRBD'

Supplementary Figure 3 – Single variant analyses in the Mendelian Randomization

The plots show the Mendelian Randomization coefficient (black dot) and 95% confidence interval (bars) for each individual variant included in the analysis. The variant analyzed is indicated on the Y axis. The red dot and line represent the results if all the variants are included.

SNP: single nucleotide polymorphism. MR: Mendelian Randomization; PD: Parkinson's disease; RBD: REM sleep behavior disorder.

Comple list of authors, including IPDGC consortium and 23andMe members:

Yuri L. Sosero,[1,2], Karl Heilbron,[3], Pierre Fontanillas,[3], Lucy Norcliffe-Kaufmann,[3], Eric Yu,[1,2], Uladzislau Rudakou,[1,2], Jennifer A. Ruskey,[2], Kathryn Freeman,[2], Farnaz Asayesh,[2], Kajsa A. Brolin,[4], Maria Swanberg,[4], Huw R Morris,[5,6], Lesley Wu,[5,6], Raquel Real,[5,6], Lasse Pihlstrøm,[7], Manuela Tan,[7], Thomas Gasser,[8], Kathrin Brockmann,[8], Hui Liu,[8], Michele T.M. Hu,[9,10], Donald G. Grosset,[11], Simon J.G. Lewis,[12], John B. Kwok,[12], Pau Pastor,[13,14], Ignacio Alvarez,[13,14], Matej Skorvanek,[15,16], Alexandra Lackova,[15,16], Miriam Ostrozovicova,[15,16], Mie Rizig,[17] , Lynne Krohn,[1,2], Ziv Gan-Or,[1,2,18], Alastair J Noyce[19],[20], Rauan Kaiyrzhanov[21], Ben Middlehurst[22], Demis A Kia[21],[23], Henry Houlden[21], Catherine S Storm[24,25], Helene Plun-Favreau[21], Peter Holmans[26], John Hardy[21], Daniah Trabzuni[21],[27], John Quinn[22], Vivien Bubb[22], Kin Y Mok[21], Kerri J. Kinghorn[28], Nicholas W Wood[21],[23], Patrick Lewis[29], Sebastian R Schreglmann[21], Ruth Lovering[24], Lea R'Bibo[21], Claudia Manzoni[29], Mie Rizig[21], Mina Ryten[21], Sebastian Guelfi[21], Valentina Escott-Price[26], Viorica Chelban[21], Thomas Foltynie[21], Nigel Williams[26], Karen E. Morrison[30], Carl Clarke[31], Kirsten Harvey[32], Benjamin M Jacobs[30], Alexis Brice[33], Fabrice Danjou[33], Suzanne Lesage[33], Jean-Christophe Corvol[33],[34], Maria Martinez[35], Claudia Schulte[36], Javier Simón-Sánchez[36], Peter Heutink[36], Patrizia Rizzu[36], Manu Sharma[37], Susanne A. Schneider[38], Mark R Cookson[39], Sara Bandres-Ciga[39], Cornelis Blauwendraat[39], David W. Craig[40], Kimberley Billingsley[39], Mary B. Makarious[39], Derek P. Narendra[41], Faraz Faghri[39],[42], J Raphael Gibbs[39], Dena G. Hernandez[39], Kendall Van Keuren-Jensen[43], Joshua M. Shulman[44], Hirotaka Iwaki[39], Hampton L. Leonard[39], Mike A. Nalls[39],[45], Laurie Robak[44], Jose Bras[46], Rita Guerreiro[46], Steven Lubbe[47], Bernabe I. Bustos[28], Timothy Troycoco[48], Steven Finkbeiner[49], Niccolo E. Mencacci[47], Codrin Lungu[50], Andrew B Singleton[39], Sonja W. Scholz[51], Xylena Reed[39], Roy N. Alcalay[52], Zbigniew K. Wszolek[53], Ryan J. Uitti[53], Owen A. Ross[53], Francis P. Grenn[39], Anni Moore[39], Vanessa Pitz[39], Guy A. Rouleau[54], Kheireddin Mufti[54], Jacobus J van Hilten[55], Johan Marinus[55], Astrid D. Adarmes-Gómez[56], Miquel Aguilar[56], Victoria Alvarez[57], Francisco Javier Barrero[58], Jesús Alberto Bergareche Yarza[59], Inmaculada Bernal-Bernal[56], Marta Blazquez[57], Marta Bonilla-Toribio[56], Juan A. Botía[60], María Teresa Boungiorno[56], Dolores Buiza-Rueda[56], Ana Cámara[61], Fátima Carrillo[56], Mario Carrión-Claro[56], Debora Cerdan[62], Jordi Clarimón[63], Yaroslau Compta[61], Monica Diez-Fairen[56], Oriol Dols-Icardo[63], Oriol de Fabregues[64], Pilar Sanz Cartagena[65], Jacinto Duarte[62], Raquel Duran[66], Francisco Escamilla-Sevilla[67], Mario Ezquerra[61], Cici Feliz[68], Manel Fernández[61], Rubén Fernández-Santiago[61], Ciara Garcia[57], Pedro García-Ruiz[68], Pilar Gómez-Garre[56], Maria Jose Gomez Heredia[69], Isabel Gonzalez-Aramburu[70], Ana Gorostidi Pagola[59], Janet Hoenicka[71], Jon Infante[70], Silvia Jesús[56], Adriano Jimenez-Escrig[72], Jaime Kulisevsky[63], Miguel A. Labrador-Espinosa[56], Jose Luis Lopez-Sendon[72], Adolfo López de Munain Arregui[59], Daniel Macias[56], Irene Martínez Torres[73], Juan Marín[63], Maria Jose Marti[61], Juan Carlos Martínez-Castrillo[72], Carlota Méndez-del-Barrio[56], Manuel Menéndez González[57], Marina Mata[74], Adolfo Mínguez[67], Pablo Mir[56], Elisabet Mondragon Rezola[59], Esteban Muñoz[61], Javier Pagonabarraga[63], Pau Pastor[56], Francisco Perez Errazquin[69], Teresa Periñán-Tocino[56], Javier Ruiz-Martínez[59], Clara Ruz[66], Antonio Sanchez Rodriguez[70], María Sierra[70], Esther Suarez-Sanmartin[57], Cesar Tabernero[62], Juan Pablo Tartari[56], Cristina Tejera-Parrado[56], Eduard Tolosa[61], Francesc Valldeoriola[61], Laura Vargas-González[56], Lydia Vela[75], Francisco Vives[66], Alexander Zimprich[76], Mathias Toft[77], Pille Taba[78], Sulev Koks[79], Sharon Hassin-Baer[80], Kari Majamaa[81], Ari Siitonen[81], Pentti Tienari[82], Njideka U. Okubadejo[83], Oluwadamilola O. Ojo[83], Nazira Zharkinbekova[84], Vadim Akhmetzhanov[85], Gulnaz Kaishybayeva[86], Altynay Karimova[86], Timothy L. Lynch[87], Stella Aslibekyan[3], Adam Auton[3], Elizabeth Babalola[3], Robert K. Bell[3], Jessica Bielenberg[3], Jonathan Bowes[3], Katarzyna Bryc[3], Ninad S. Chaudhary[3], Daniella Coker[3], Sayantan Das[3], Emily DelloRusso[3], Sarah L. Elson[3], Nicholas Eriksson[3], Teresa Filshtein[3], Pierre Fontanillas[3], Will Freyman[3], Zach Fuller[3], Chris German[3], Julie M. Granka[3], Karl Heilbron[3], Alejandro Hernandez[3], Barry Hicks[3], David A. Hinds[3], Ethan M. Jewett[3], Yunxuan Jiang[3], Katelyn Kukar[3], Alan Kwong[3], Yanyu Liang[3], Keng-Han Lin[3], Bianca A. Llamas[3], Matthew H. McIntyre[3], Steven J. Micheletti[3], Meghan E. Moreno[3], Priyanka Nandakumar[3], Dominique T.

Nguyen[3], Jared O'Connell[3], Aaron A. Petrakovitz[3], G. David Poznik[3], Alexandra Reynoso[3], Shubham Saini[3], Morgan Schumacher[3], Leah Selcer[3], Anjali J. Shastri[3], Janie F. Shelton[3], Jingchunzi Shi[3], Suyash Shringarpure[3], Qiaojuan Jane Su[3], Susana A. Tat[3], Vinh Tran[3], Joyce Y. Tung[3], Xin Wang[3], Wei Wang[3], Catherine H. Weldon[3], Peter Wilton[3], Corinna D. Wong[3]

Affiliations

1. Department of Human Genetics, McGill University, Montréal, QC, Canada.
2. The Neuro (Montreal Neurological Institute-Hospital), McGill University, Montréal, QC, Canada.
3. 23andMe, Inc., Sunnyvale, California, USA.
4. Lund University, Translational Neurogenetics Unit, Department of Experimental Medical Science, Lund, Sweden.
5. Department of Clinical and Movement Neurosciences, UCL Queen Square Institute of Neurology, University College London, UK
6. UCL Movement Disorders Centre, University College London, London, UK
7. Department of Neurology, Oslo University Hospital, Oslo, Norway
8. Department of Neurodegeneration at Hertie Institute for Clinical Brain Research, Tuebingen, Germany
9. Oxford Parkinson's Disease Centre (OPDC), University of Oxford, Oxford, United Kingdom.
10. Nuffield Department of Clinical Neurosciences, University of Oxford, Oxford, United Kingdom.
11. Institute of Neurological Sciences, Queen Elizabeth University Hospital, Glasgow, United Kingdom
12. Parkinson’s Disease Research Clinic, Brain and Mind Centre, School of Medical Sciences, University of Sydney, Australia
13. Unit of Neurodegenerative diseases, Department of Neurology, University Hospital Germans Trias i Pujol and The Germans Trias i Pujol Research Institute (IGTP) Badalona, Barcelona, Spain
14. Department of Neurology, Hospital Universitari Mutua de Terrassa, Barcelona, Spain
15. Department of Neurology, Pavol Jozef Šafárik University in Košice, Slovakia
16. Department of Neurology, University Hospital of L. Pasteur, Kosice, Slovakia
17. UCL Queen Square Institute of Neurology, London, UK
18. Department of Neurology and Neurosurgery, McGill University, Montreal, QC, Canada.
19. Preventive Neurology Unit, Wolfson Institute of Preventive Medicine, QMUL, London, UK
20. Department of Molecular Neuroscience, UCL, London, UK
21. Department of Molecular Neuroscience, UCL Institute of Neurology, London, UK
22. Institute of Translational Medicine, University of Liverpool, Liverpool, UK
23. UCL Genetics Institute, London, UK
24. Department of Clinical Neuroscience, University College London, London, UK
25. Department of Clinical and Movement Neurosciences, UCL Queen Square Institute of Neurology, London, UK
26. MRC Centre for Neuropsychiatric Genetics & Genomics, Cardiff University School of Medicine, Cardiff, UK
27. Department of Genetics, King Faisal Specialist Hospital and Research Centre, Riyadh, Saudi Arabia
28. Institute of Healthy Ageing, Research Department of Genetics, Evolution and Environment, University College London, London, UK
29. University of Reading, Reading, UK
30. Faculty of Medicine, University of Southampton, UK
31. University of Birmingham, Birmingham, UK and Sandwell and West Birmingham Hospitals

NHS Trust, Birmingham, UK
32. UCL School of Pharmacy, UK
33. Institut du Cerveau et de la Moelle épinière, ICM, Inserm U 1127, CNRS, UMR 7225, Sorbonne Universités, UPMC University Paris 06, AP-HP, Pitié-Salpêtrière Hospital, Paris, France
34. Centre d'Investigation Clinique Pitié Neurosciences CIC-1422, AP-HP, Pitié-Salpêtrière Hospital, Paris, France
35. INSERM UMR 1220, Paul Sabatier University, Toulouse, France
36. Department for Neurodegenerative Diseases, Hertie Institute for Clinical Brain Research, University of Tübingen, and DZNE, German Center for Neurodegenerative Diseases, Tübingen, Germany
37. Centre for Genetic Epidemiology, Institute for Clinical Epidemiology and Applied Biometry, University of Tubingen, Germany
38. Department of Neurology, Ludwig-Maximilians-University Munich, München, Germany
39. Laboratory of Neurogenetics, National Institute on Aging, Bethesda, MD, USA
40. Department of Translational Genomics, Keck School of Medicine, University of Southern California, Los Angeles, USA
41. Inherited Movement Disorders Unit, National Institute of Neurological Disorders and Stroke, Bethesda, MD, USA
42. Department of Computer Science, University of Illinois at Urbana-Champaign, Urbana, IL, USA
43. Neurogenomics Division, TGen, Phoenix, AZ, USA
44. Departments of Neurology, Neuroscience, and Molecular & Human Genetics, Baylor College of Medicine, Houston, Texas, USA
45. Data Tecnica International, Glen Echo, MD, USA
46. Center for Neurodegenerative Science, Van Andel Research Institute, Grand Rapids, Michigan, USA
47. Ken and Ruth Davee Department of Neurology and Simpson Querrey Center for Neurogenetics, Northwestern University Feinberg School of Medicine, Chicago, IL, USA
48. National Institutes of Health, USA
49. Departments of Neurology and Physiology, University of California, San Francisco; Gladstone Institute of Neurological Disease; Taube/Koret Center for Neurodegenerative Disease Research, San Francisco, CA, USA
50. National Institutes of Health Division of Clinical Research, NINDS, National Institutes of Health, Bethesda, MD, USA
51. Neurodegenerative Diseases Research Unit, National Institute of Neurological Disorders and Stroke, Bethesda, MD, USA
52. Department of Neurology, College of Physicians and Surgeons, Columbia University Medical Center, New York, NY, USA, Taub Institute for Research on Alzheimer's Disease and the Aging Brain, Columbia University Medical Center, New York, NY, USA
53. Department of Neurology, Mayo Clinic Jacksonville, FL, USA
54. Montreal Neurological Institute and Hospital, Department of Neurology & Neurosurgery, Department of Human Genetics, McGill University, Montréal, QC, Canada
55. Department of Neurology, Leiden University Medical Center, Leiden, Netherlands
56. Instituto de Biomedicina de Sevilla (IBiS), Hospital Universitario Virgen del Rocío/CSIC/Universidad de Sevilla, Seville, Spain
57. Hospital Universitario Central de Asturias, Oviedo, Spain
58. Hospital Universitario San Cecilio de Granada, Universidad de Granada, Spain
59. Instituto de Investigación Sanitaria Biodonostia, San Sebastián, Spain
60. Universidad de Murcia, Murcia, Spain
61. Hospital Clinic de Barcelona, Spain

62. Hospital General de Segovia, Segovia, Spain
63. Memory Unit, Department of Neurology, IIB Sant Pau, Hospital de la Santa Creu i Sant Pau, Universitat Autònoma de Barcelona, Barcelona, and Centro de Investigación Biomédica en Red en Enfermedades Neurodegenerativas (CIBERNED), Madrid, Spain
64. Movement Disorders Unit, Neurology Department, University Hospital Vall d'Hebron, Barcelona, Spain
65. Hospital de Mataro, Consorci Sanitari del Maresme, Mataro, Spain
66. Centro de Investigación Biomédica, Universidad de Granada, Granada, Spain
67. Hospital Universitario Virgen de las Nieves, Instituto de Investigación Biosanitaria de Granada, Spain
68. Instituto de Investigación Sanitaria Fundación Jiménez Díaz, Madrid, Spain
69. Hospital Universitario Virgen de la Victoria, Malaga, Spain
70. Hospital Universitario Marqués de Valdecilla-IDIVAL, Santander, Spain
71. Institut de Recerca Sant Joan de Déu, Barcelona, Spain
72. Hospital Universitario Ramón y Cajal, Madrid, Spain
73. Department of Neurology, Instituto de Investigación Sanitaria La Fe, Hospital Universitario y Politécnico La Fe, Valencia, Spain
74. Department of Neurology, Hospital Universitario Infanta Sofía, Madrid, Spain
75. Department of Neurology, Hospital Universitario Fundación Alcorcón, Madrid, Spain
76. Department of Neurology, Medical University of Vienna, Austria
77. Department of Neurology, Oslo University Hospital, Oslo, Norway
78. Department of Neurology and Neurosurgery, University of Tartu, Tartu, Estonia
79. Centre for Molecular Medicine and Innovative Therapeutics, Murdoch University, Murdoch, Perth, Western Australia; The Perron Institute for Neurological and Translational Science, Nedlands, Perth, Western Australia
80. The Movement Disorders Institute, Department of Neurology and Sagol Neuroscience Center, Chaim Sheba Medical Center, Tel-Hashomer, Ramat Gan, Israel; Sackler Faculty of Medicine, Tel Aviv University, Tel Aviv, Israel
81. Institute of Clinical Medicine, Department of Neurology, University of Oulu, Oulu, Finland; Department of Neurology and Medical Research Center, Oulu University Hospital, Oulu, Finland
82. Clinical Neurosciences, Neurology, University of Helsinki, Helsinki University Hospital, Helsinki, Finland
83. University of Lagos, Lagos State, Nigeria
84. South Kazakhstan Medical Academy, Shymkent, Kazakhstan
85. Astana Medical University, Astana, Kazakhstan
86. Scientific and Practical Center "Institute of Neurology named after Smagul Kaishibayev", Almaty, Kazakhstan
87. The Dublin Neurological Institute at the Mater Misericordiae University Hospital, Dublin, Ireland & School of Medicine and Medical Science, University College Dublin, Dublin, Ireland
